# SON-1210 - a novel bifunctional IL-12 / IL-15 fusion protein that improves cytokine half-life, targets tumors, and enhances therapeutic efficacy

**DOI:** 10.3389/fimmu.2023.1326927

**Published:** 2023-12-20

**Authors:** John K. Cini, Susan Dexter, Darrel J. Rezac, Stephen J. McAndrew, Gael Hedou, Rich Brody, Rukiye-Nazan Eraslan, Richard T. Kenney, Pankaj Mohan

**Affiliations:** ^1^ Sonnet BioTherapeutics, Inc., Princeton, NJ, United States; ^2^ Latham Biopharm Group, Inc., Elkridge, MD, United States; ^3^ Sonnet BioTherapeutics, CH S.A., Geneva, GE, Switzerland; ^4^ InfinixBio, Inc., Athens, OH, United States; ^5^ Invivotek, LLC., Hamilton, NJ, United States

**Keywords:** interleukin-12, interleukin-15, cancer, tumor microenvironment, immunomodulation, Fully human albumin binding (FHAB) domain, interferon gamma, immunotherapy

## Abstract

**Background:**

The potential synergy between interleukin-12 (IL-12) and IL-15 holds promise for more effective solid tumor immunotherapy. Nevertheless, previous clinical trials involving therapeutic cytokines have encountered obstacles such as short pharmacokinetics, limited tumor microenvironment (TME) targeting, and substantial systemic toxicity.

**Methods:**

To address these challenges, we fused single-chain human IL-12 and native human IL-15 in *cis* onto a fully human albumin binding (F_H_AB) domain single-chain antibody fragment (scFv). This novel fusion protein, IL12-F_H_AB-IL15 (SON-1210), is anticipated to amplify the therapeutic impact of interleukins and combination immunotherapies in human TME. The molecule was studied *in vitro* and in animal models to assess its pharmacokinetics, potency, functional characteristics, safety, immune response, and efficacy.

**Results:**

SON-1210 demonstrated robust binding affinity to albumin and exhibited the anticipated *in vitro* activity and tumor model efficacy that might be expected based on decades of research on native IL-12 and IL-15. Notably, in the B16F10 melanoma model (a non-immunogenic, relatively “cold” tumor), the murine counterpart of the construct, which had mouse (m) and human (h) cytokine sequences for the respective payloads (mIL12-F_H_AB-hIL15), outperformed equimolar doses of the co-administered native cytokines in a dose-dependent manner. A single dose caused a marked reduction in tumor growth that was concomitant with increased IFNγ levels; increased Th1, CTL, and activated NK cells; a shift in macrophages from the M2 to M1 phenotype; and a reduction in Treg cells. In addition, a repeat-dose non-human primate (NHP) toxicology study displayed excellent tolerability up to 62.5 µg/kg of SON-1210 administered three times, which was accompanied by the anticipated increases in IFNγ levels. Toxicokinetic analyses showed sustained serum levels of SON-1210, using a sandwich ELISA with anti-IL-15 for capture and biotinylated anti-IL-12 for detection, along with sustained IFNγ levels, indicating prolonged kinetics and biological activity.

**Conclusion:**

Collectively, these findings support the suitability of SON-1210 for patient trials in terms of activity, efficacy, and safety, offering a promising opportunity for solid tumor immunotherapy. Linking cytokine payloads to a fully human albumin binding domain provides an indirect opportunity to target the TME using potent cytokines in *cis* that can redirect the immune response and control tumor growth.

## 1 Introduction

Interleukin-12 (IL-12) is a multifunctional cytokine that regulates cell-mediated innate and adaptive immune responses and orchestrates potent anticancer effects, either alone or synergistically with other cytokines (1). IL-12 primes natural killer (NK) cells and T-helper type 1 (Th1) cells to secrete IFNγ, reactivate and enhance the survival of memory CD4^+^ T cells, help differentiate CD8^+^ T cells, and upregulate IL-15, IL-18, and IL-2-receptor expression while decreasing the levels of Treg cells and their impact on immunosuppression (2). IL-12 can also inhibit neovascularization due to induction of IFNγ (3) via upregulation of angiostatin (4) or suppression of the vascular endothelial growth factor receptor 3 (VEGFR3) (5). IL-15 shares many biological properties with IL-12, including upregulation of IL-12 beta receptor expression and maturation, as well as NK and memory CD8^+^ T-cell proliferation and activation. The prolonged survival of CD8^+^ memory T cells enhances the duration of tumor immune surveillance for months and potentially even years.

The combination of IL-15 with other cytokines in *cis* can enhance antitumor activity compared to either cytokine alone, which correlates with the synergistic upregulation of each cytokine’s receptors, resulting in a marked induction of IFNγ (6). The capacity of dendritic cells (DCs) to secrete IL-12 and present IL-15 is crucial. Both IL-12 and IL-15 mediate NK-cell activation by DCs in human lymphoid organs (7). Cultured DCs from either blood or spleen primarily stimulate CD56^bright^CD16^-^ NK cells, which are enriched in secondary lymphoid tissue. Blocking of IL-12 abolished the DC-induced IFNγ secretion by NK cells *in vitro*, whereas membrane-bound IL-15 on DCs is essential for NK cell proliferation and survival. DCs colocalize with NK cells *in vivo* in the T-cell areas of lymph nodes. CD40 ligation promotes the highest IL-15 surface presentation during maturation of the DCs and leads to the strongest NK cell proliferation. This causes increased IFNγ production, which increases MHC on DCs, making antigen presentation more efficient. Combining IL-15 with IL-12 drives the generation of more NK maturation, creating highly functional NK cells *in vitro*, resulting in >70% positivity for CD16 and/or KIR within 2 weeks after infusion into mice (6). There is a clear potential for further SON-1210 combination *in vivo* with checkpoint inhibitors or cell-based therapy. The interplay between activation of NK cells, CD4^+^ T cells, and CD8^+^ T cells and local delivery of cytokines and αPD-L1 therapeutics to immune cell-containing *in vitro* melanoma tumors was recently modeled (8). Both NK cells and CD8^+^ T cells were shown to be necessary for tumor cell killing and CD4^+^ T-cell activation was reduced without NK cells. Delivery of IL-15/IL-15Rα to tumor cells effectively mediated anti-tumor activity and sensitized the tumor microenvironment (TME) for therapy with αPD-L1 therapeutics, mainly by impacting NK cells.

The effectiveness of cancer immunomodulators depends on the interplay between the physical properties of the drug and the TME, including permeability, resident immune cell activation or suppression of inhibition, retention time within the tumor, and serum pharmacokinetic (PK) properties (9–11). Smaller proteins (< 100 kDa) favor improved penetration into solid tumors, whereas longer protein half-lives (up to 21 days vs. minutes to hours) can extend the duration of tumor exposure (12). We devised a strategy to prolong cytokine PK half-life (t_1/2_) and target the TME by proposing the use of an albumin single-chain antibody fragment (scFv), which is a fully human albumin-binding (F_H_AB) domain (13, 14). The F_H_AB domain exploits the physiological recycling of albumin by binding to the neonatal Fc receptor (FcRn) in a manner similar to FcRn recycling of IgG (15), leading to increased half-life. Notably, a more important consideration is that albumin facilitates targeted delivery of the F_H_AB to the TME due to its marked accumulation in tumors by enhanced penetration and retention (16).

**Figure 1:**
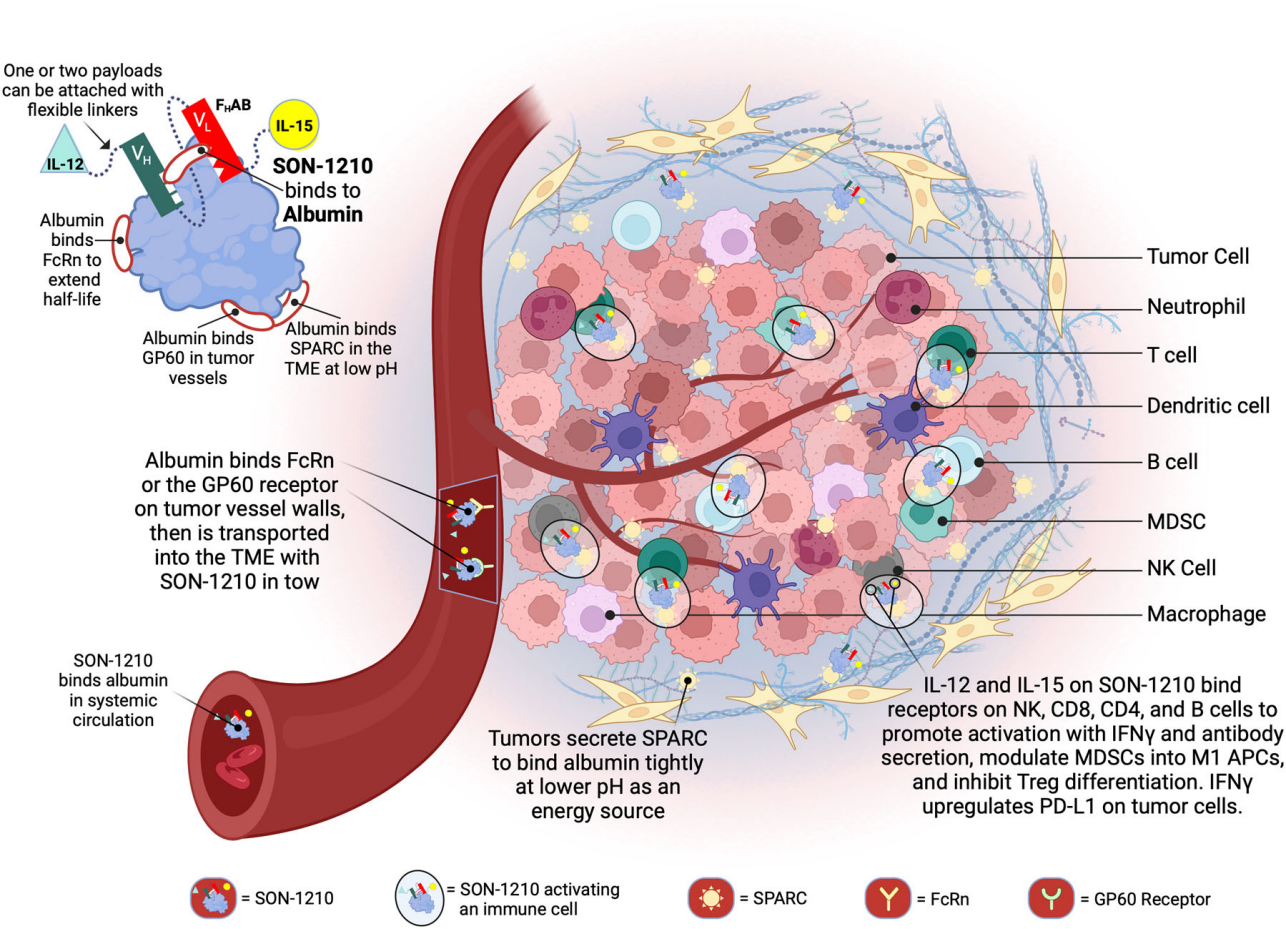
Schematic Representation of SON-1210 and its Mechanism of Action. The F_H_AB molecule on the top left consists of a scFv heavy chain (V_H_ in green) linked using ([GGGGS]_3_) to a light (V_L_ in red) chain that comprises an albumin-binding domain. Therapeutic payloads can be fused to each side of the central construct using flexible linkers ([GGGGS]_5_), as indicated by the light green triangle and yellow circle (14). The conformation shown here represents SON-1210 (comprising single-chain hIL-12 and native hIL-15 sequence linked to the F_H_AB for monkey or human use) or mIL12-F_H_AB-hIL15, consisting of the mouse cognate of IL-12 and the human version of IL-15 for rodent studies. The F_H_AB construct can interact with albumin from all 3 species, which binds systemically to FcRn, to share albumin’s extended PK. The entire complex can be carried into the tumor tissue through the bloodstream, where the FcRn and GP60 receptors are upregulated, to be transported across the endothelium into the acidic TME. Once there, the albumin binds tightly in dynamic equilibrium via interaction with SPARC, which is overexpressed in the TME. The IL-12 and IL-15 cytokine domains can activate resident immune cells and recruit more cells, upregulating expression of IFNγ from NK, CD4^+^, and CD8^+^ T cells, which then upregulates PD-L1 on tumor cells and antibody production from B cells. Created with BioRender.com.

To find an appropriate binding moiety, a fully human single chain antibody fragment phage library (XOMA, Emeryville, CA) with > 1 × 10^11^ variable heavy and variable kappa/lambda light chain diversities was screened using a number of criteria to isolate an anti-albumin scFv. The desired characteristics used to identify the best F_H_AB include (i) high-affinity binding to human, mouse, and cynomolgus serum albumin; (ii) low double-digit nanomolar binding at physiological pH 7.2 and binding at a lower pH 5.8, which is characteristic of the acidic TME; (iii) selection of an anti-albumin epitope that preserves the binding site for FcRn, thus preventing renal clearance while retaining the benefit of FcRn-mediated recycling of albumin for extended PK; and (iv) preservation of the binding sites on albumin for albondin (GP60) and the “secreted protein acidic and rich in cysteine” (SPARC) tumor antigens, to enable extended TME accumulation and retention (17). SON-1210 is a novel drug candidate based on the F_H_AB platform (**Figure 1**) that includes single-chain IL-12 and native IL-15 attached via flexible linkers to the amino and carboxy termini of the F_H_AB domain, respectively, to create IL12-F_H_AB-IL15. Linkers were designed to minimize potential steric hindrance of the attached protein(s). This design enables the extended half-life and activity of both cytokines, while bridging certain synergies of innate and adaptive tumor immunity.

Mechanistic proof of concept was originally demonstrated using a 4T1 mouse mammary tumor model positive for high expression of TGFβ (13). Both the isolated F_H_AB and anti-TGFβ linked to the F_H_AB efficiently targeted the implanted tumor and were present from 0.5 to 24 h after injection (**Supplementary Material, Figure 1.2**). However, the anti-TGFβ scFv, which strongly binds to TGFβ with a K_d_ of 10 nM, targeted the tumor alone, but was only present in lysates for up to 4 h, suggesting that it had diffused out of the tumor at later time points. Murine IL-12 was similarly linked to F_H_AB to target murine B16F10 tumors and enhance its efficacy compared with native IL-12. This approach provided at least a 30-fold improvement in the therapeutic index (18). The monofunctional human IL12-F_H_AB (SON-1010) is currently being tested clinically (19, 20).

IL-12 and IL-15 are strong inducers of antitumor activity and have been evaluated in numerous clinical studies (21–23). Combinatorial approaches using stimulatory cytokines, checkpoint inhibitors, chemotherapy, and/or radiation therapy are known to improve overall survival of patients with cancer (24, 25). However, recombinant interleukins have had limited clinical success owing to their short circulating half-life, inefficient TME targeting, and requirement for frequent dosing, leading to substantial systemic toxicities (26). To address these challenges, the F_H_AB platform provides immunomodulators in a mono- or bifunctional format by employing the scFv to bind albumin, which improves their PK profiles and enhances TME targeting. Albumin binds efficiently to proteins such as FcRn, GP60, and SPARC, which are overexpressed in many solid tumors, to provide concentration and retention of a drug molecule that is bound to an albumin molecule in the TME. IL-12 also transforms pro-tumor M2 myeloid-derived suppressor cells (MDSCs) into inflammatory M1 antigen presenting cells (APCs) leading to recovery of their macrophage function, thus turning “cold” tumors into “hot” tumors (27). The consequence of binding to albumin and being transported to the tumor tissue has the potential to be more effective than native IL-12 at lower doses, which further decreases the risk of toxicity and results in a broader therapeutic index. The significant cancer therapy potential of the combination of IL-12 and IL-15 promises to take advantage of IL-12’s ability to prime innate/adaptive immune responses, while IL-15 can boost and maintain an antitumor response (28, 29). In this way, the synergies associated with each of the biological functions of these cytokines can be leveraged, namely, the ability of IL-12 to rapidly activate innate and adaptive immune responses and that of IL-15 to potently stimulate the proliferation of CD4^+^ T cells and maintain memory CD8^+^ T cells (6, 26, 30).

## 2 Materials and methods

### 2.1 Protein production and reagents

The murine version of SON-1210, which has mouse (m) and human (h) cytokine sequences in the respective payload positions (mIL12-F_H_AB-hIL15), was produced in CHO cells using shake flasks. The monofunctional mIL12-F_H_AB, hIL12-F_H_AB, and hIL15-F_H_AB molecules, as well as mIL-12, hIL-12, hIL-15, and their His-tagged versions were produced in the same way (31). The SON-1210 clinical manufacturing process was performed using continuous perfusion during a 15-day manufacturing process. Purification was accomplished using multiple chromatography steps, resulting in a Good Manufacturing Process (GMP) product quality that is suitable for human use. All molecules were formulated in histidine-based buffer containing trehalose, DTPA, and polysorbate 20 at pH 7.3. The diluent used as the negative control was identical to the formulation buffer. Some recombinant human IL-12 (hIL-12), murine IL-12 (mIL-12), and human IL-15 (hIL-15) reference material and reagents used in these studies were purchased from Peprotech (Cat# 200-12, Cat# 200-15, respectively), and R&D Systems (Cat# 419-ML-010/CF).

### 2.2 Characterization of the F_H_AB platform

To develop IL12-F_H_AB-IL15 (SON-1210) as a human therapeutic, the compound must be tested for safety and efficacy in at least one relevant animal species. The first requirement of establishing an animal model is demonstrating that the F_H_AB domain binds to that animal’s serum albumin. This binding event was investigated by Surface Plasmon Resonance (SPR) to show relative affinity, comparing human and monkey serum albumin *in vitro*. The mIL12-F_H_AB and hIL15-F_H_AB constructs also demonstrated that mice can be used to show the effects of the extended PK due to binding of albumin *in vivo*. The second requirement for assessing the relevance of an animal species is that the cytokine portions of the target compound, single-chain hIL-12 and native hIL-15, bind to and activate the IL-12 and IL-15 receptors on the relevant animal’s PBMCs. This requirement was tested by treating PBMCs from the selected animal species with hIL12-F_H_AB-hIL15 in at least one potency assay for each cytokine component. Human and cynomolgus monkey PBMCs were purchased from IQ Biosciences (Cat# IQB-PBMC103 and IQB-MnPB102, respectively).

#### 2.2.1 Surface plasmon resonance assay of F_H_AB binding

Selection of an appropriate species for testing of hIL12-F_H_AB-hIL15 was done using SPR to evaluate its binding to albumin at physiologic pH 7.4 and at an acidic pH of 5.8, to approximate conditions in the TME. Species studied included rat (Sigma Cat# A6414), Syrian hamster (Bio-world Cat# 22070085-1, further purified by InfinixBio), canine (Abcam Cat# ab119814), or macaque (Athens Research Cat# 16-16-011202-CM) serum albumin, compared to the degree of binding to human serum albumin (Abcam Cat# ab205808) (RSA, HamSA, CSA, MSA, and HSA, respectively). Assay details are included in the **Supplementary Materials Section 1.1**.

#### 2.2.2 hIL12-F_H_AB-hIL15 (SON-1210) binding to Albumin

The solution dissociation constant (K_d_) of SON-1210 was determined for binding to various concentrations of albumin (**Supplementary Materials Sections 1.2**) (32). Briefly, to determine the solution K_d_ for binding to albumin, ELISA plates were coated with the appropriate species albumin. Test samples that contained 2 nM SON-1210 were incubated with different concentrations of either HSA or MSA in buffer, or no albumin as control. Aliquots were transferred to the ELISA plate, incubated for 1h, then for another hour after adding an anti-human IL-12p70 biotinylated detection antibody (ThermoFisher Cat#CUST77216). After washing, samples were incubated with Streptavidin-HRP (ThermoFisher 21130), washed four times, and visualized with TMB (Sera Care Cat#5120-0083) for 10 minutes before quenching with 0.05 mL 1 M HCl and reading the plate at 450 nm. The K_d_ value was determined at each concentration of added albumin.

#### 2.2.3 SPARC binding to HSA or HSA : IL12-F_H_AB

To determine the solution K_d_ of HSA binding to SPARC, ELISA plates were coated with HSA. At the same time samples containing Biotinylated (B)-SPARC were incubated for 1h in polypropylene microtiter plates with different concentrations of HSA (3000 nM –>3 nm; 2 fold dilutions) in in pH 6.0 PBS + 0.05% Tween 20. Aliquots of the samples containing B-SPARC and HSA were then transferred to the coated plates and incubated for 1h at room temperature. After washing, the plates were incubated for 1h with 0.25 µg/mL Streptavidin-HRP (ThermoFisher). The plates were then visualized with TMB (Sera Care) for 20 minutes before quenching with 0.05 mL 1 M HCl and reading the plate at 450 nm. (further detail in **Supplementary Material Section 1.3**).

To show that SPARC binds to the HSA : IL12-F_H_AB complex in pH 6.0 PBS + 0.05% Tween 20, ELISA plates were coated with HSA and then incubated with mixtures of B-SPARC (0.15 nM) + IL12-F_H_AB (1500 nM, 750 nM, or 0 nM). The concentrations of IL12-F_H_AB used in this experiment were shown to saturate the HSA on the plate in a prior study. After washing three times, the binding of B-SPARC to IL12-F_H_AB on the plate was visualized by incubation with 0.25 µg/mL Streptavidin-HRP as described above.

#### 2.2.4 Naïve mouse pharmacokinetics

To evaluate PK, three C57BL/6 mice for each time point were administered a single intravenous (IV) dose of 5 µg mIL12-F_H_AB-His, hIL15-F_H_AB-His, mIL-12-His, or hIL-15-His. Quantitation of each compound in serum was performed with an ELISA, based on consistent detection of a histidine (His) tag to avoid potential interference from the native cytokines. Plates were coated overnight with 6x-His Epitope Tag Antibody (Pierce Cat#His.H8) at 2-8°C, then washed, and sample dilutions added for 1 h incubation at room temperature. Rabbit anti-6-His biotinylated secondary antibody (Bethyl Cat# A190-114B) was then added to the wells for 1 h at room temperature, followed by streptavidin poly-HRP (ThermoScientific Cat# 21140) for 30 minutes at 37°C. The reaction was stopped with H_2_SO_4_ and the plates were read at 450nm.

#### 2.2.5 Lymphoblast proliferation assay for IL-12 activity

The lymphoblast proliferation assay is a functional assessment of the ability of IL-12 to stimulate the proliferation of PHA-activated T lymphoblasts (“PHA blasts”). Lymphoblast formation is triggered by the treatment of healthy human donor PBMCs (Precision for Medicine) with phytohemagglutinin P (PHA-P) for 4 days, with the addition of IL-2 during the final 24 hrs, before the proliferation assay is done. Once the PHA blasts are formed, their proliferation can be stimulated by the presence of functional recombinant hIL-12 (R&D Cat# 219-IL-005) or mIL12-F_H_AB-hIL15 over a period of 48 hours using the CellTiter Aqueous One Solution Cell Proliferation kit (Promega Cat# G3580), which includes media supplemented with 5% FBS. Cell proliferation is quantitated by the addition of CellTiter-Glo^®^ Luminescent Reagent (Promega Cat# G7571) using a microplate reader with luminometer attached. A 4-parameter logistic curve is generated to assess cell proliferation and to determine EC_50_ values.

#### 2.2.6 IFNγ secretion by PHA blasts

IL-12 or IL-15 functional activity can be assessed by their ability to induce the secretion of IFNγ from PHA blasts. Lymphoblasts were prepared and stimulated as described above. After incubation with hIL12-F_H_AB (SON-1010), hIL12-F_H_AB-hIL15, or recombinant hIL-12, the culture medium was then processed for the detection of IFNγ with a human IFNγ ELISA. Increased IFNγ secretion by hIL12-F_H_AB-hIL15, as compared to that induced by a hIL-12 control standard, indicates that an IL-12 functional product was present in the culture.

#### 2.2.7 CTLL-2 proliferation assay for IL-15 activity

This functional assay is based on the ability of hIL-15 to stimulate the proliferation of the murine cytotoxic T-cell line CTLL-2 (ATCC Cat#TIB-214). CTLL-2 cells are able to proliferate in response to either human or mouse IL-15. This proliferative response can be measured under controlled tissue culture conditions for IL-15 products over a 48 hour period. Cell proliferation was quantitated by the addition of CellTiter 96^®^ Aqueous One Solution Cell Proliferation Assay Reagent (MTS) and a microplate reader with luminometer attached. A 4-parameter logistic curve is generated to assess cell proliferation and to determine EC_50_ values.

#### 2.2.8 Tumor accumulation of radiolabeled mIL12-F_H_AB

The B16F10 model (Section 2.4) was used to assess biodistribution of mIL12-F_H_AB-His compared to mIL-12-His in a preliminary study (Charles River Laboratories, Mattawan, MI). Both the recombinant molecules used in this study were expressed in CHO cells and purified to > 95% by SDS-PAGE and SE-HPLC (Abzena, Cambridge, UK). The final products demonstrated activity by the HEK-Blue assay, which confirms STAT4 phosphorylation as discussed below (Section 2.3.2). We showed that the mIL12-F_H_AB-His binds HSA and mouse serum albumin using SPR with a K_d_ ranging from 30-50 nM.

Both mIL12-F_H_AB-His and mIL-12-His products were then radiolabeled with technitium-99m (^99m^Tc), and were then purified using standard methods (33) to make [^99m^Tc]-mIL-12-His and [^99m^Tc]-mIL12-F_H_AB-His. Groups of mice with or without tumors were injected with 1 µg/g of each product containing ≤ 200µCi per animal IV, once the tumors had reached about 200 mm^3^, and were then imaged with a Mediso nanoSPECT/CT scanner over the next 96 h. Image processing and analysis was performed using VivoQuant™ Software.

### 2.3 Characterization of SON-1210

#### 2.3.1 Physical characteristics of SON-1210

Samples from two GMP batches were run using a 4-20% Bis-Tris gel by sodium dodecyl sulfate-polyacrylamide gel electrophoresis (SDS-PAGE) compared to molecular weight (MW) markers at Enzene Biosciences, Ltd. (Pune, India). Purity was assessed by size exclusion-high pressure liquid chromatography (SE-HPLC). Charge heterogeneity was evaluated by imaged capillary isoelectric focusing (iCIEF) using standard techniques (34).

#### 2.3.2 Potency measurement of the IL-12 and IL-15 moieties in SON-1210

Activation of IL-12 and IL-15 receptors by their natural ligands induces phosphorylation of STAT4 and STAT5, respectively. HEK-Blue is a reporter gene assay that can be used to measure the potency of IL-12 and IL-15 as independent entities in the bifunctional molecule, SON-1210 (**Supplementary Material Section 1.5**). HEK-Blue IL-12 cells express a STAT4-inducible secreted embryonic alkaline phosphatase (SEAP) reporter gene triggered by the binding of IL-12 to its receptor (35). The cytokines hIL-2 and hIL-15 are closely related and share the heterodimeric CD122 (IL-2Rβ)/CD132 (IL-2Rγ) receptor to deliver signals to the target cells. Therefore, to determine the relative bioactivity of hIL-15, SON-1210 was tested using HEK-Blue IL-2 reporter cells that express STAT5-inducible SEAP upon binding of IL-15 to the IL-2 receptor (36). Both assays were done in growth medium containing 10% heat-inactivated FBS.

#### 2.3.3 Comparative stimulation of IFNγ production

The functionality of the IL-12 and IL-15 moieties of SON-1210 was evaluated by measuring IFNγ production from both human and monkey PBMCs *in vitro* after prior stimulation of the cells with PHA-L for 72 hours, then IL-2 for 24 h. Washed cell aliquots were incubated with the SON-1210, IL-12 (Peprotech, Cat# 200-12), or media alone for 48h, and the level of IFNγ was determined by ELISA (Human IFNγ High Sensitivity ELISA Kit [Abcam, Cat# ab46048]; Monkey IFNγ ELISA PRO Kit [Mabtech, Cat# 3421M-1HP-1]) (**Supplementary Material Section 1.6**).

#### 2.3.4 T-cell proliferation

The ability of SON-1210 to promote T- and NK-cell proliferation was studied in monocyte-depleted human PBMCs to avoid intrinsic IL-12 interference. Cells were stimulated with PHA-L for three days, then IL-2 was added for another 24 h. The cells were washed and incubated with the media containing SON-1210 or IL-15 (with or without anti-IL-15 antibody [R&D Systems, Cat# MAB247]) for 48 h and cell proliferation was determined using a luminescence assay. As SON-1210 and IL12-F_H_AB share the same IL-12 domain, the proliferation effect was compared using equimolar concentrations of the two stimulators or was neutralized with an anti-IL-12 antibody (R&D Systems, Cat# MAB219) (**Supplementary Material Section 1.7**). Based on these results, 25 µg/mL of anti-IL-12 antibody was found to be sufficient to block the function of hIL12-F_H_AB at 50 pM or lower.

### 2.4 Safety and efficacy in B16F10 tumor-bearing mice

The antitumor efficacy of mIL12-F_H_AB-hIL15 was assessed in a subcutaneous (SC) B16F10 syngeneic mouse melanoma model using C57BL/6 mice by measuring the evolution of tumor volume and survival over time. Animals were randomly grouped (**Table 1**) with sample size based on prior experience. Treatments were initiated when the mean tumor volume reached approximately 100 mm^3^, seven days after inoculation of 0.2 × 10^6^ B16F10 melanoma cells (**Supplementary Material Section 2**). The effect of IV administration of a single dose of mIL12-F_H_AB-hIL15 at several dose levels or three doses at 5 µg/dose was compared with that of the vehicle, as well as equimolar administration of recombinant mIL-12, hIL-15, their combination, or mIL12-F_H_AB. The Tumor-Bearing Placebo group received the melanoma cells and placebo administration, while the Non-Tumor Bearing (naïve) group received no treatment and was used for hematology and clinical chemistries on day 0. Treatments were administered by IV injection in 200 μL of 0.02% Tween 20 in PBS on the day of dosing (day 0).

**Table 1:** Design of the B16F10 mouse efficacy study.

Treatment	Single Dose	Dose 3x (Day 0, 2, 4)	Terminal Bleeds (n=)			FACS (n=)	Efficacy (n=)	Total Mice (N=173)
			Day 0	Day 3	Day 8			
**Tumor-Bearing Placebo**	–	–	4	4	4	4	8	24
**mIL12-F_H_AB-hIL15**	1 µg	-	-	5	5	-	8	18
**mIL12-F_H_AB-hIL15**	5 µg	-	-	5	5	5	8	23
**mIL12-F_H_AB-hIL15**	10 µg	-	-	5	5	-	8	18
**mIL12 reference** **(based on 5ug dose)**	3 ug	-	-	4	4	-	-	8
**hIL15 reference (based on 5ug dose)**	0.8 ug	-	-	5	5	-	-	10
**mIL12 + hIL-15 references**	3 µg + 0.8 µg	-	-	4	4	5	8	21
**mIL12-F_H_AB-hIL15**	-	5 µg	-	5	5	5	8	23
**mIL12-F_H_AB**	5 µg	-	-	5	5	5	8	23
**Non-Tumor Bearing Placebo (Naïve Mice)**			5	–	–	–	–	5

C57BL/6 females were randomly assigned to treatment groups as described. Control groups are shown in white, purple, and blue. The number of mice per group and analysis conducted are shown on each of the columns. Acclimation was 2 weeks; age at initiation was 10-11 weeks. Animals were excluded if their tumors were < 100 mm^3^ or > 150 mm^3^ on the day of dosing. None of the animals entered onto the study were excluded from the analysis. Tumors for FACS were harvested on Day 3. Efficacy was assessed with measurements of tumor volume on even days until sacrifice. Animals were euthanized if the tumors exceeded 1800 mm^3^ or body weight loss was >20% as indicated in the IACUC approval.

Body weight was used as an early indicator of toxicity, along with hematological and clinical chemical analyses. The levels of IFNγ, IL-10, IL-12p70, IL-1β, IL-2, IL-4, IL-5, IL-6, KC/gro (IL-8-related protein in rodents), and TNFα were determined in sera using an electrochemiluminescence panel (Meso Scale Discovery, Cat# K15048D).

Tumor samples were collected for FACS analysis when the average tumor size was approximately 250 mm^3^ on day three (**Supplementary Material Figure 2**). Single cell suspensions from freshly collected spleens and tumors were prepared by transferring individual tissues into gentleMACS C Tubes containing 5 ml of RPMI, which were placed onto a gentleMACS tissue dissociator (Miltenyi Biotec). The single cell suspensions were filtered through Falcon 100 µm nylon filters and centrifuged. The staining panel included markers for live/dead, CD45, TCR-beta, CD8, CD4, CD25, FoxP3, IFNγ, CD49b, F4/80, CD206, CD11b, CD11c and MCHII.

### 2.5 Toxicokinetic evaluation of repeated SON-1210 dosing in non-human primates

Hamster, rat, dog, and macaque cells were screened to determine the best species to use for toxicology studies with an *in vitro* assessment of binding and biopotency. Macaque cells were the only ones that responded similarly to the human control cells. A preliminary dose-ranging study was done with SON-1210 in non-human primates (NHPs). A total of 28 male and female Mauritian cynomolgus macaques received SON-1210 given subcutaneously (SC) at 15.6 to 125.0 µg/kg, either one time on day 0 or twice, on days 0 and 15. This study was intended to establish the maximum tolerated dose (MTD).

In the subsequent Good Laboratory Practice (GLP) study, the safety, toxicology, and toxicokinetic (TK) attributes of SON-1210 were evaluated in 32 male and female cynomolgus macaque NHPs (**Table 2** and **Supplementary Material Section 3**). The dose range of 15.625, 31.25, and 62.5 µg/kg was targeted to assess the maximum pharmacological effect, using 3 dose levels plus a control group, with sample size based on prior experience. Three males and 3 females were enrolled as the main group (studied for 6 weeks), with 2 additional animals of each sex at the highest dose and controls, added as a recovery group (studied for 11 weeks). The protocol and procedures involving the care and use of animals in the study were reviewed and approved by Charles River Institutional Animal Care and Use Committee (IACUC) before conduct. This study was aligned with the ICH and FDA guidelines for preclinical assessment of biopharmaceutical products.

**Table 2:** Design of the GLP toxicology study in NHPs.

Group No.	Test Article	Dose Level Days 1, 15, & 29 (µg/kg/dose)	Dose Volume^a^ (mL/kg)	Main Study	Recovery Study	Total NHPs (N=32)
				No. of Animals	No. of Animals	
**1**	Vehicle	0	2	3 M and 3 F	2 M/2F	10
**2**	SON-1210	15.6	2	3 M and 3 F	–	6
**3**	SON-1210	31.2	2	3 M and 3 F	–	6
**4**	SON-1210	62.5	2	3 M and 3 F	2 M/2F	10

Male and female NHPs were assigned to groups upon animal transfer based on established pairs but were randomized separately. Animals in poor health or at extremes of body weight range were not assigned to groups. A minimum acclimation period of 10 days was allowed between animal transfer and the start of treatment to accustom the animals to the laboratory environment. Before the initiation of dosing, one animal was rejected from the study due to hematology findings and was replaced by a spare animal. Vehicle control was the histidine formulation buffer for SON-1210. Dosing occurred once every 14 days on Days 1, 15, and 29.

a Based on the most recent body weight measurement. No., number; -, not applicable.

Following three SC administrations on days 1, 15, and 29, animals were followed closely for clinical observations, body weight, and food consumption. Animals were routinely monitored ophthalmologically and by electrocardiography, along with comprehensive hematological and clinical chemistry assessments. Necropsy included gross dissection, organ weights, and histopathology. Bioanalytical samples were taken at various points during the study for cytokines, immunophenotyping, anti-drug antibody (ADA), and toxicokinetics (TK) analysis. The cytokines assessed included IFNγ, TNFα, IL-6, IL-8, IL-10, and IL-1β. ADA (IgG or IgM) was quantified in sera in two stages, starting with a screening assay followed by a confirmatory assay.

SON-1210 was quantified for TK analysis using a validated IL-12/IL-15 combination assay and was made specific by first capturing the molecule with an IL-15 domain-specific antibody (ThermoFisher, Cat# 88-7620-88), and then detecting the quantity of captured material with a biotinylated IL-12 domain-specific antibody (Thermofisher, Cat# CUST77216). Background levels of monkey IL-12 and IL-15 were negligible, so were not considered to interfere with the assay. The upper and lower limits of quantitation (ULOQ and LLOQ) for the assay were determined to be 400 pg/mL and 12 pg/mL, respectively. LLOQ results were estimated at 6 pg/mL for graphing, while the NCA analysis treated these results as zero before dosing and as missing after dosing. Samples were diluted into the quantifiable range if the initial result was above the ULOQ. TK parameters were estimated using non-compartmental analysis (Pheonix WinNonlin). Further details are included in the **Supplementary Material Section 3**.

### 2.6 Statistical analysis

All data are presented as mean ± SEM. A two-way analysis of variance (ANOVA) was performed with a between-subject variable of treatment and a repeated measurement factor of time. Tumor growth curves were evaluated using the ANOVA and Dunnett’s multiple comparison test. The Gehan-Breslow-Wilcoxon test was applied to survival curves. One-way ANOVA was applied to each cytokine response and safety laboratory test. All calculations were performed using R (R Foundation for Statistical Computing) and the GraphPad (Prism Software).

## 3 Results

### 3.1 Development and characterization of the F_H_AB platform

A schematic representation of SON-1210 is shown in the **Figure 1** inset, along with the relevant binding sites on albumin. The mechanism of delivery of the IL12-F_H_AB-IL15:albumin complex to the tumor, along with its retention and activation of immune cells in the TME, is described in the rest of **Figure 1**. The studies described below required production of both the human and murine versions of the bifunctional molecule that is shown, as well as the monofunctional molecules (hIL12-F_H_AB [aka SON-1010], mIL12-F_H_AB, and hIL15-F_H_AB).

**Figure 2:**
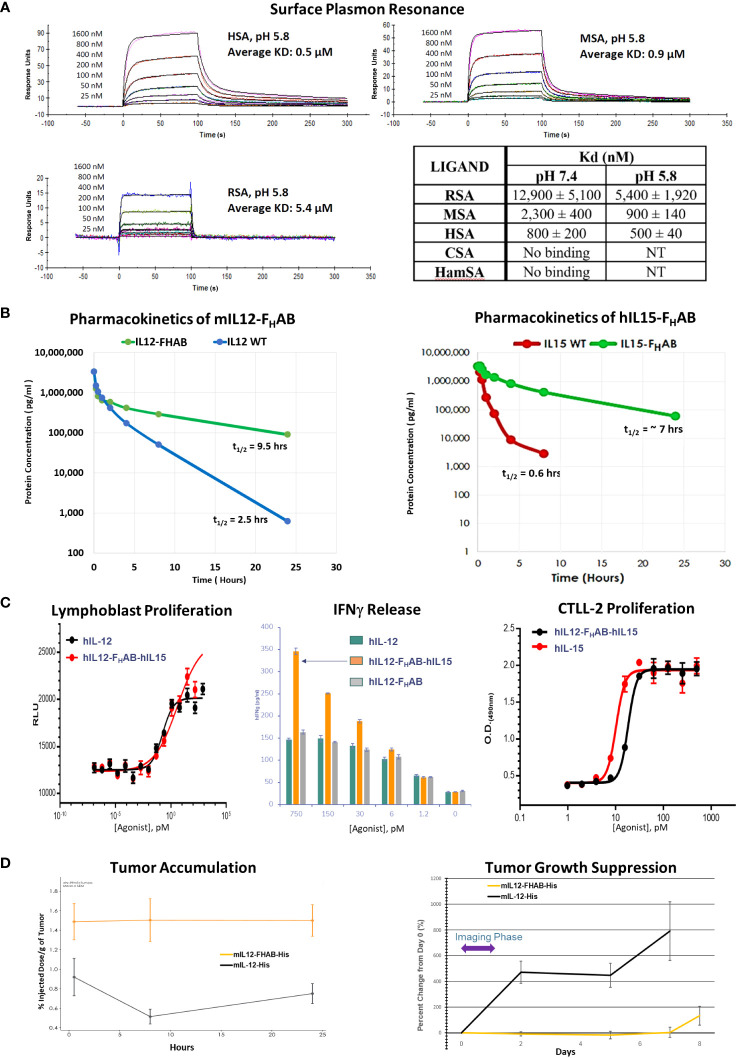
Characterization of the FHAB Platform. **(A)** Surface plasmon resonance curves at pH 5.8 of species-specific serum albumin binding to hIL12-F_H_AB-hIL15 (SON-1210). The K_d_ is the dissociation constant; a lower K_d_ indicates stronger binding affinity. Abbreviations: RSA = rat serum albumin, MSA = monkey serum albumin, HSA = human serum albumin, CSA = canine serum albumin, HamSA = hamster serum albumin. **(B)** Pharmacokinetic assessment of the mIL12-F_H_AB concentration by ELISA in the serum of naïve mice (left panel) after a single IV injection compared with injection of mIL-12. The same approach using hIL15-F_H_AB compared with hIL-15 (right panel). **(C)** A lymphoblast assay of human PBMCs was used to assess relative proliferation (left panel), comparing hIL-12 with hIL12-F_H_AB-hIL15 *in vitro*. Supernatant IFNγ released from PHA blasts following stimulation with varying concentrations of hIL-12, hIL12-F_H_AB (SON-1010), or hIL12-F_H_AB-hIL15 (center panel). IL-15 bioactivity was studied using proliferation of the murine cytotoxic T-cell line CTLL-2 (right panel) after exposure to various concentrations of hIL-15 or hIL12-F_H_AB-hIL15. **(D)** A biodistribution study designed to show tumor accumulation was done in B16F10 tumor-bearing or control mice after injection of [^99m^Tc]-mIL12-F_H_AB or [^99m^Tc]-mIL12, once the tumors had reached ~200 mm^3^. The mice were imaged by SPECT/CT over 24 hours. Tumor growth was measured over the next 8 days.

Binding of the F_H_AB domain to albumin was evaluated using species-specific responses in several types of assays. We first showed that the species most closely resembling human binding of hIL12-F_H_AB-hIL15 was cynomolgus macaque by SPR both at physiologic conditions (pH 7.4), and more importantly at conditions that mimic the extravascular space in the TME (pH 5.8) (**Figure 2A** and **Supplementary Material Figure S1**). Rat serum albumin binding of hIL12-F_H_AB-hIL15 at both conditions was much weaker, while Syrian hamster and canine serum albumin samples showed no binding. Thus, the macaque was selected as the best model for further comparisons with potential human use of hIL12-F_H_AB-hIL15.

The affinity of two molecules can also be studied by measuring the binding and unbinding reactions of receptor (R) and ligand (L) molecules in solution. This is expressed as an association constant (K_a_) or more typically as its inverse, the dissociation constant (K_d_). The solution K_d_ for the binding of hIL12-F_H_AB-hIL15 to HSA and MSA was studied at pH 7.4 and pH 6.2 (the method is described in **Supplementary Material Section 1.2**). Binding to HSA revealed K_d_ values of 60 ± 30 nM and 80 ± 10 nM, respectively. Binding to MSA showed K_d_ of 400 ± 200 nM and 600 ± 300 nM at pH 7.4 and 6.2, respectively. Thus, the binding of hIL12-F_H_AB-hIL15 to HSA was approximately seven times stronger than its binding to MSA at both pH 7.4 and 6.2.

In contrast, binding of HSA to SPARC in conditions resembling the TME was even tighter, with a K_d_ of 10 nM at pH 6.0 (the method is described in **Supplementary Material Section 1.3**), whereas minimal binding was detected at pH 7.2 by ELISA. The solution binding constant of B-SPARC to HSA:hIL12-F_H_AB could not be measured, but the binding of B-SPARC to immobilized HSA and immobilized HSA:hIL12-ABD was similar. This suggests that once the complex is delivered to the TME by FcRn- or GP60-based shuttling of receptor-bound albumin across the tumor endothelium, the F_H_AB : HSA complex is retained in that space by binding to SPARC in a dynamic equilibrium with slow release (17).

The *in vivo* half-lives of monofunctional mIL12-F_H_AB (**Figure 2B**, left panel) and hIL15-F_H_AB (**Figure 2B**, right panel) were higher compared to their native counterparts, mIL-12 and hIL-15. The half-lives of mIL-12 and hIL-15 in mouse serum improved by 3.8- and 11.6-fold, respectively, when the cytokine was linked to F_H_AB. These preclinical studies were felt to reflect the platform adequately, so a PK study of the bifunctional hIL12-F_H_AB-hIL15 molecule will wait for the first clinical study.

The next step was to confirm that human IL-12 and human IL-15 bind to and activate the IL-12 and IL-15 receptors on the relevant animal’s PBMCs. Activation of the IL-12 receptor was studied using PHA lymphoblasts made from human PBMCs. Cell proliferation following exposure to hIL12-F_H_AB-hIL15 closely resembled stimulation by recombinant hIL-12 (**Figure 2C**, left panel). Activation of the IL-15 receptor was studied using the murine cytotoxic T-cell line CTLL-2. Proliferation assessed with multiple concentrations of hIL12-F_H_AB-hIL15 was nearly as effective as hIL-15 (**Figure 2C**, right panel). Finally, the physiologic impact was assessed by IFNγ production using PHA blasts. While the effect of hIL12-F_H_AB was nearly the same as recombinant hIL-12, hIL12-F_H_AB-hIL15 stimulated the production of IFNγ more efficiently at concentrations above 6 pM (**Figure 2C**, center panel).

In addition, while biodistribution studies have not yet been conducted with the IL12-F_H_AB-IL15 molecule, a study has been completed using the monofunctional IL12-F_H_AB in mice. Tumor accumulation of the mIL12-F_H_AB platform molecule and mIL-12 was measured by radiolabeling both His-tagged molecules with ^99m^Tc. After IV administration of each labeled molecule into a cohort of B16F10 tumor-bearing or control C57BL/6 mice, the [^99m^Tc]-mIL12-F_H_AB molecule accumulated 1.7- to 3.1-fold higher over a 0.5- to 24-hour period in the tumor, compared to the [^99m^Tc]-mIL12 control (**Figure 2D**, left panel). The radiolabel analysis was terminated at 24 hours due to the rapid decay of the label (the t_1/2_ of ^99m^Tc is 6 hours). Tumors were followed with measurements until sacrifice showing prolonged suppression of growth by the mIL12-F_H_AB molecule (**Figure 2D**, right panel).

### 3.2 Characterization of SON-1210

#### 3.2.1 Physical characteristics of SON-1210

**Figure 3:**
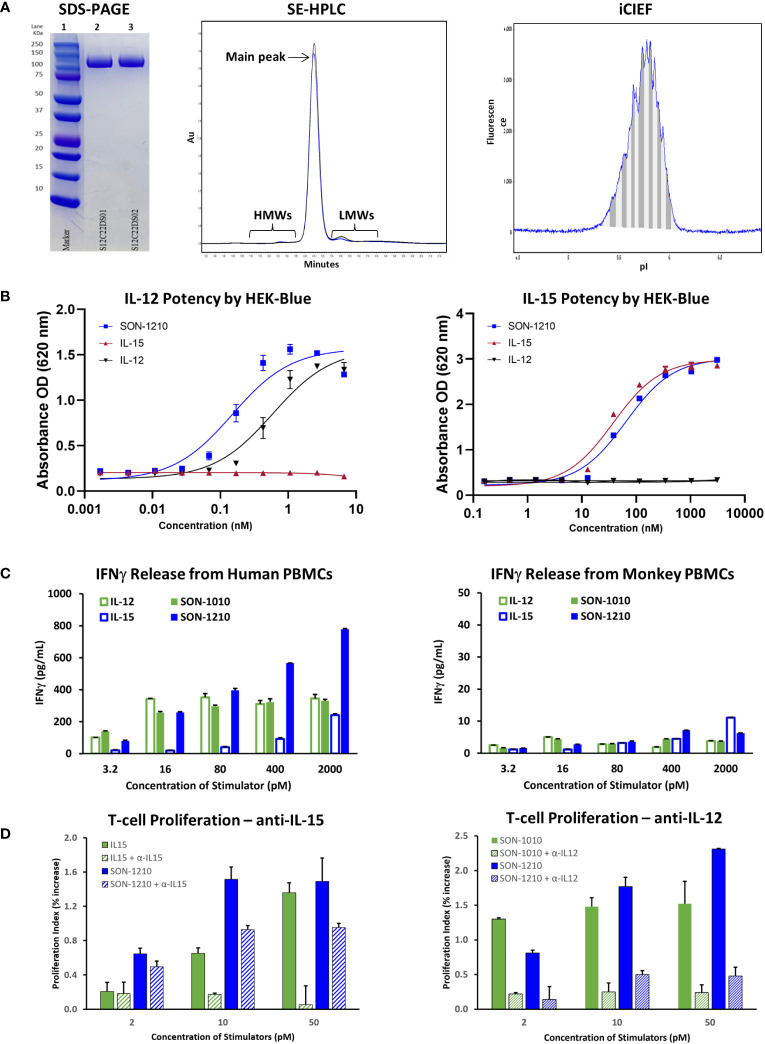
Characterization of SON-1210. **(A)** Non-reducing SDS-PAGE of SON-1210 (left panel). Samples from two GMP drug substance batches (lanes 2 and 3) were run on a 4-20% Bis-Tris gel with MW markers (lane 1). The purity of two GMP batches of SON-1210 was evaluated by size exclusion HPLC (center panel), which shows > 97% monomeric product in each. Minor high MW (HMWs) and low MW (LMWs) peaks flank the main peak at about 14.3 m. Charge heterogeneity of SON-1210 was assessed by imaged capillary isoelectric focusing (iCIEF) (right panel); a representative sample is shown. **(B)** The potency of the IL-12 (left panel) and IL-15 (right panel) moieties of SON-1210 was assessed using HEK-Blue 12 or HEK-Blue 2 cells. **(C)** Production of IFNγ by SON-1210 or hIL12-F_H_AB (SON-1010) in the supernatant of cultured PBMCs from both humans (left panel) and monkeys (right panel). **(D)** Assessment of the individual moieties of SON-1210 to stimulate the proliferation of PHA-L and IL-2 stimulated, monocyte-depleted human PBMCs (to preactivate the T-cells) in the presence or absence of anti-IL15 or anti-IL12 antibodies. hIL-15 is used as the control for SON-1210 (left panel) and SON-1010 is the control in the right panel.

Samples from two GMP drug substance (DS) batches of hIL12-F_H_AB-hIL15 (SON-1210) were evaluated by non-reduced SDS-PAGE. While the theoretical MW is 99 kDa, the observed MW was about 115 kDa due to glycosylation (**Figure 3A**, left panel). The purity of the products was next studied by SE-HPLC. The main peak resolved at about 14.3 m and each was > 97% pure, with minor high MW (HMWs) and low MW (LMWs) peaks flanking the main peak (**Figure 3A**, center panel). Charge heterogeneity was assessed by iCIEF. Charge variations of biomolecules are common and can generally include those caused deamidation, formation of N-terminal pyroglutamate, aggregation, isomerization, sialylated glycans, antibody fragmentation, and glycosylation (34). A representative graph from the analysis of one of the DS batches shows minor heterogeneity with an isoelectric point (pI) of 5.3 to 6.0 (**Figure 3A**, right panel), compared to the theoretical pI of 5.3 to 5.4. These results were all within their prespecified acceptance criteria ranges, allowing further processing to make SON-1210 drug product (DP).

#### 3.2.2 Potency by HEK-Blue assay

HEK-Blue cells expressed a STAT4-inducible SEAP reporter that was triggered by the binding of IL-12 to the IL-12 receptor (**Figure 3B**, left panel). SON-1210 exhibited approximately three-fold higher potency (EC_50 = _0.16 nM) than native hIL-12 (EC_50 = _0.48 nM).

The activity of the IL-15 portion of SON-1210 was measured using a HEK-Blue IL-2 reporter assay (**Figure 3B**, right panel). This revealed IL-15 activity of SON-1210 of approximately 60% of the IL-15 positive control with EC_50_ values of 57.4 pM versus 33.1 pM, respectively.

#### 3.2.3 IFNγ production by PBMCs

In human PBMCs, SON-1210 induced clear dose-dependent stimulation of IFNγ release. The induction of IFNγ by SON-1210 was higher than that elicited by the native IL-12 control at doses higher than 16 pM and higher than that of native IL-15 at all doses (**Figure 3C**, left panel). This result is consistent with our previous findings in tumor B16F10 mouse models, showing that mIL12-F_H_AB stimulates more IFNγ than mIL-12 at higher dose levels (18). SON-1210 also generated higher IFNγ levels than hIL12-F_H_AB (SON-1010), presumably because of the addition of IL-15 in *cis* to the former molecule.

In monkey PBMCs, SON-1210 induced higher levels of IFNγ than hIL-12 controls (except at the lowest doses) and hIL-15 controls (except at the highest dose tested) (**Figure 3C**, right panel). While they are the only type of animal PBMCs that responded measurably to human constructs, monkey cells are expected to respond to human cytokines less efficiently, owing to species sequence differences of the cytokines and receptors (37).

#### 3.2.4 T-cell proliferation assay for the contribution of IL-12 and IL-15

SON-1210, IL-12, and IL-15 should all trigger a dose-dependent proliferative effect in human PBMCs. The ability of SON-1210 to stimulate T- and NK-cell proliferation was compared to that of hIL12-F_H_AB (SON-1010) and hIL15-F_H_AB in human PBMCs using specific HEK-Blue assays to qualitatively establish the activity of each moiety individually. Both IL-12 and IL-15 have been shown to induce PHA-L-stimulated T- and NK-cell proliferation (38). In the presence of anti-IL-15 antibody at 50 µg/mL, the function of hIL-15 was blocked; SON-1210 induced stronger proliferation of human PBMCs than hIL-12, with or without anti-IL-15. This indicates that the IL-12 portion of SON-1210 remained active in this assay (**Figure 3D**, left panel). SON-1210 also stimulated T-cell proliferation despite blocking the function of the IL-12 portion of SON-1210 with a 25 µg/mL anti-IL-12 antibody, indicating that the IL-15 portion of SON-1210 was also active in this assay (**Figure 3D**, right panel). The effective stimulation range for the IL-12 portion of SON-1210 was found to be 2-10 pM, and the effective stimulation range for the IL-15 portion of SON-1210 was 2-50 pM.

### 3.3 Safety and efficacy in tumor-bearing mice

**Figure 4:**
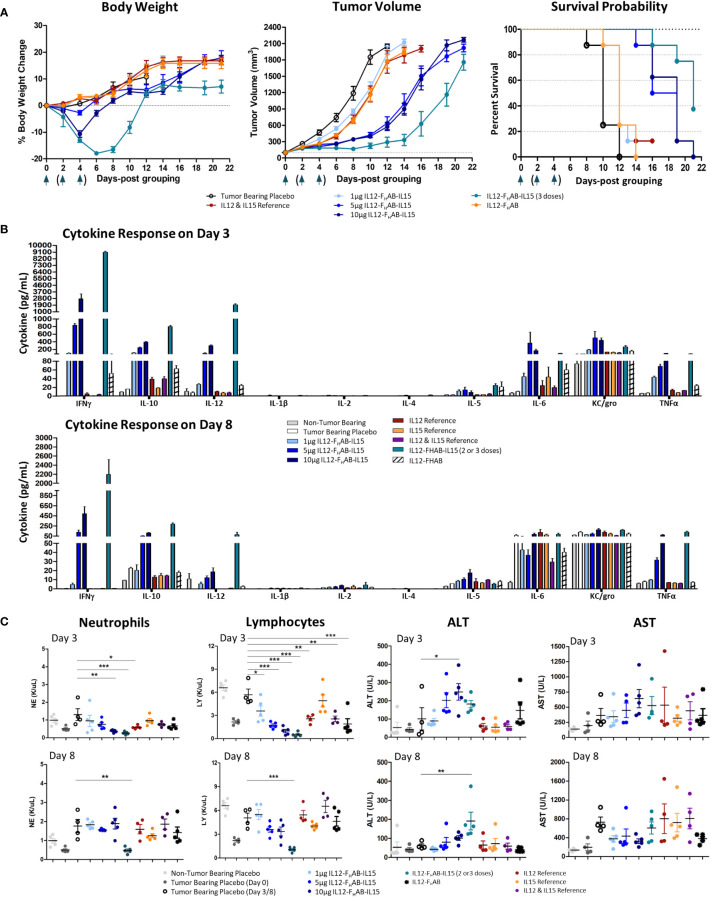
Safety and Efficacy of mIL12-FHAB-hIL15 in the Mouse. **(A)** Mice were implanted with B16F10 murine melanoma cells via SC inoculation and the tumors were allowed to grow to approximately 100 mm^3^ before the start of the study. Groups of mice were dosed once on day 0 as shown in the legend or three times on days 0, 2, and 4 (arrows). Body weight gain was expressed as a percentage change compared with the initial weight of each animal (left panel). The tumor volume was measured using a caliper on alternate days until euthanasia. All groups were significantly different from the tumor-bearing placebo control by day 6 (Bonferroni’s post-test: *p* < 0.001) (center panel). The survival probability is displayed over time as a function of treatment (log-rank test for the trend: *p* < 0.01) (right panel). **(B)** Blood cytokine levels measured on days 3 and 8 after a single dose of each compound on day 0, or after 2 doses (measured on day 3, top panel) or 3 doses (measured on day 8, bottom panel) of mIL12-F_H_AB-hIL15. **(C)** Hematological analyses evaluating neutrophil (1^st^ panel) and lymphocyte (2^nd^ panel) counts on days 3 and 8. Clinical chemistry analysis for alanine (ALT, 3^rd^ panel) and aspartate aminotransferase (AST, 4^th^ panel) levels measured on days 3 (top panels) and 8 (bottom panels). Dunnett’s multiple comparison test: * (*p* < 0.5), ** (*p* < 0.01), *** (*p* < 0.001).

The B16F10 syngeneic SC mouse melanoma non-immunogenic “cold” tumor model (39) was used to evaluate the antitumor efficacy of mIL12-F_H_AB-hIL15. No clinical signs or deaths were reported during the study. Body weight of the mice (n=8/group) was measured three times per week until a tumor volume of 1800 mm^3^ was achieved (**Figure 4A**, left panel). A two-way ANOVA with multiple comparisons showed that treatment with a single dose of mIL12-F_H_AB-hIL15 resulted in moderate weight loss on day 4 (placebo vs. 10 μg mIL12-F_H_AB-hIL15, p < 0.05) that began to rebound by day 6. Despite a significant reduction in body weight after the highest single dose treatment, animals recovered on day 8 and did not differ substantially from the tumor-bearing placebo group. Three doses of mIL12-F_H_AB-hIL15 resulted in a more dramatic weight loss on days 4-10 (placebo vs. 5 μg × 3 qod of mIL12-F_H_AB-hIL15, p < 0.0001) that fully recovered by Day 12. This degree of toxicity did not require sacrifice of any mice but may be a limiting factor in doing repeat-dose studies in this model.

Compared to placebo-treated mice, mIL12-F_H_AB-hIL15 mice (n=8/group) showed slower tumor growth in a dose-dependent manner (**Figure 4A**, center panel). A single dose of 5 µg was fully effective, whereas a single dose of 10 µg did not further slow the tumor volume increase. The 3x group showed an even more effective response, with tumor growth delayed until day 14. All groups treated with mono- or bifunctional cytokine(s) linked to F_H_AB showed significant growth inhibition, starting on day 4. Bonferroni’s post test revealed significant differences (p < 0.001) in tumor volume between the tumor-bearing placebo group and all cytokine groups starting on day 6.

Finally, a time-to-event efficacy approach in the mice (n=8/group) revealed an increase in survival following mIL12-F_H_AB-hIL15 treatment (**Figure 4A**, right panel), with 1 µg inducing 12-day median survival, whereas 10 µg induced 19-day median survival, compared to 10 days in the tumor-bearing placebo mice. Thus, there was a clear dose-dependent effect of mIL12-F_H_AB-hIL15 treatment on survival (log-rank test for the trend: *p* < 0.01). The median survival with a single mIL12-F_H_AB-hIL15 dose of 5 µg was 18.5 days, which was prolonged to 21 days after 3 doses.

Analysis of the pharmacodynamic (PD) cytokine response 3 days after dosing (**Figure 4B**) showed that mIL12-F_H_AB-hIL15 increased IFNγ, IL-10, IL-12, IL-6, and TNFα levels in a dose-dependent manner compared to the tumor-bearing placebo group, with no evidence of cytokine release syndrome. There was a substantial increase in IFNγ levels with a single dose of mIL12-F_H_AB-hIL15 to over 2000 pg/mL at 3 days, whereas mild increases were observed in other cytokines. Two doses of 5 µg increased the peak response to almost 9000 pg/mL. By day 8, the cytokine response pattern was sustained but generally dampened, with maximal IFNγ levels returning to 500 pg/mL after a single dose or 2100 pg/mL after three doses of mIL12-F_H_AB-hIL15 at 5 µg. However, TNFα levels remained elevated.

Hematological and chemistry examination 3 days after IV dosing showed that mIL12-F_H_AB-hIL15 treatment led to a dose-dependent reduction in neutrophils and lymphocytes on day 3 (**Figure 4C**, left panels) compared to the corresponding levels in placebo-treated tumor-bearing mice. RBCs were stable on day 3 in all groups but fell significantly in the high-dose and 3x groups by day 8 (**Supplementary Material, Figure S3**). Platelet counts also fell to half the pretreatment levels at all doses on day 3 and then rebounded by day 8 in all but the 3x group. Blood chemistry showed a mild, dose-dependent, transient increase in alanine aminotransferase (ALT) levels by day 3 with mIL12-F_H_AB-hIL15 that were significant at the 10 µg dose (but not after 2 doses of 5 µg), with no significant increases in aspartate aminotransferase (AST) at either day 3 or day 8 (**Figure 4C**, right panels).

**Figure 5:**
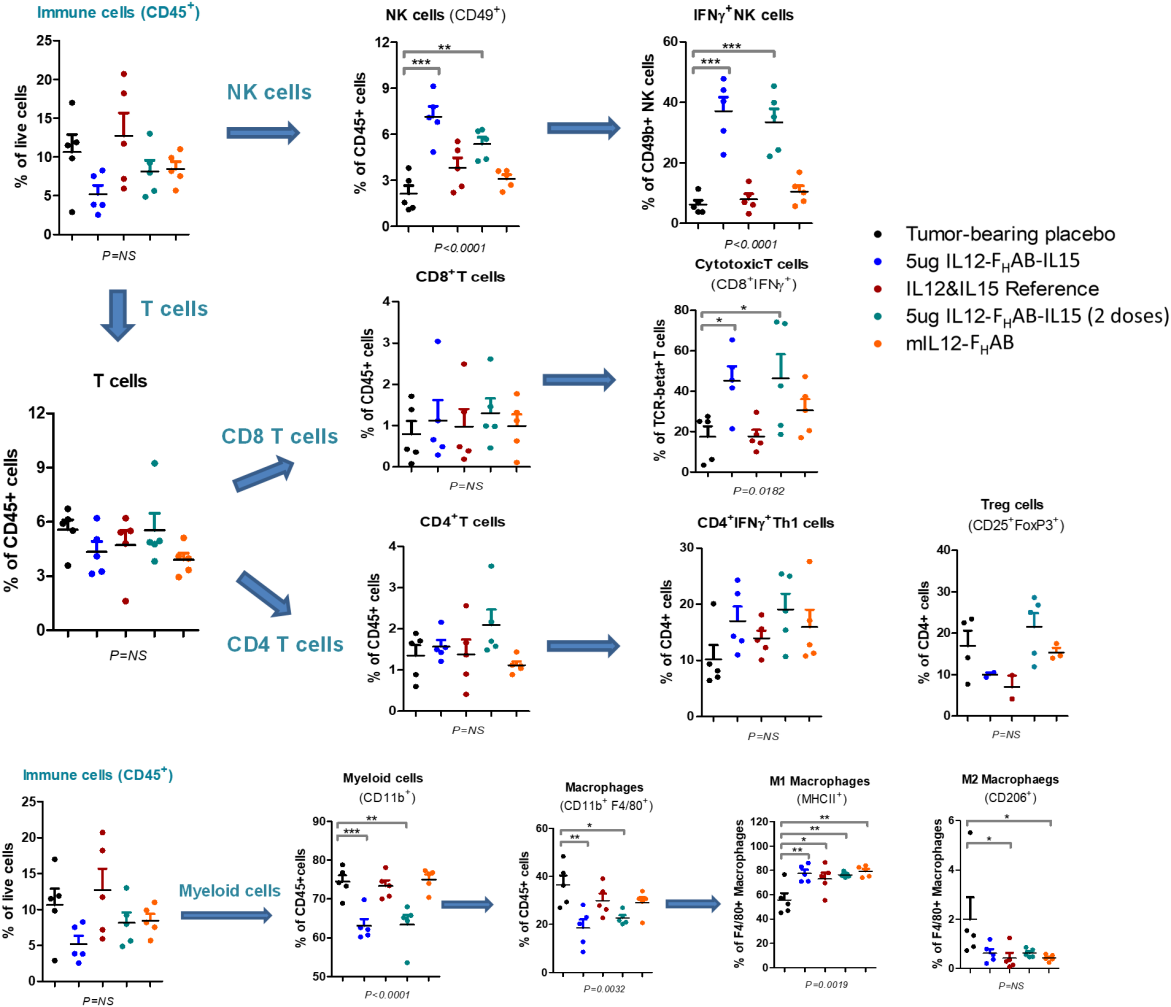
Cell Subsets in B16F10 Tumors by FACS Analysis. Flow cytometry was used to analyze the cellular distribution of tumors harvested on day 3 after one or two doses of mIL12-F_H_AB-hIL15. NK cells were gated on CD45^+^TCR^-^CD49b^+^, CD4 T cells on CD45^+^TCR^+^CD49b^-^CD4^+^, CD8 T cells on CD45^+^TCR^+^CD49b^-^CD8^+^, Tregs on CD45^+^TCR^+^CD49b^-^CD4^+^CD25^+^FoxP3^+^, and M1 vs. M2 macrophages on CD45^+^TCR^-^CD11b^+^F4/80^+^MHCII^+^CD206^-^ vs. CD45^+^TCR^-^CD11b^+^F4/80^+^MHCII^-^CD206^+^. P-values are shown as * (*p* < 0.5), ** (*p* < 0.01), *** (*p* < 0.001). The FACS gating strategy and more results are presented in **Supplementary Material Section 2**. The FACS analysis was done with FlowJo software (BD Biosciences).

FACS analysis was performed on tumors cells harvested on day 3 to compare the pattern of cellular responses after treatment with one dose of 1, 5, or 10 µg (or two doses of 5 µg) mIL12-F_H_AB-hIL15 versus a single dose of equimolar amounts of the co-administered cytokines as a control (**Figure 5**). A 3-fold increase in NK cells over placebo was observed, with a 2-fold increase compared to cytokine controls. Activated IFNγ^+^ NK cells increased 5-fold compared to cytokine controls. Activated (CD8^+^ IFNγ^+^) cytotoxic T cells (CTLs) were detected in the tumor upon treatment with mIL12-F_H_AB-hIL15 at a 2.5-fold increase compared with the control. As expected (40), activated CD4 Th1 cells increased and Treg cells decreased, but there was no significant change. The number of myeloid cells and total macrophages was reduced in the mIL12-F_H_AB-hIL15 dose group, along with a significant increase in M1 macrophages, whereas M2 macrophages were slightly decreased. A second dose of mIL12-F_H_AB-hIL15 showed the same pattern as a single dose for activated NK cells. In all cases of treatment or doses, no significant effect was observed on dendritic cells (See **Supplementary Material Section 2** for T-cell gating strategy [**Table S1**], along with tumor [**Figure S5**] and spleen [**Figure S6**] sample results).

### 3.4 Toxicokinetic evaluation of SON-1210 in NHPs

In the preliminary non-GLP study that was designed to establish the MTD, SON-1210 was well tolerated up to 62.5 µg/kg in single and repeat subcutaneous doses in both males and female NHPs. Mild decreases were seen in the white blood cell count (WBC) and lymphocytes at day 3 with recovery to predose levels by days 7 to 10 for all groups. There was a mild increase in total bilirubin at day 3 with resolution by day 7, along with a mild increase in aspartate transaminase (AST) at day 7 with resolution to predose levels by day 14 or sooner for all groups. IFNγ was increased at 48 to 96 h in all groups; no responses or abnormal increases were seen in the other cytokines measured (IL-1β, IL-6, IL-8, IL-10, or TNFα). B, T, and NK cells were all decreased at day 3 by FACS analysis with general recovery by day 7 to 10 in all groups. Severe clinical abnormalities were seen in the non-GLP study after a dose of 125 µg/kg in both females at days 8 and 10, warranting euthanasia of those two NHPs. Thus, the 62.5 µg/kg dose was considered as the MTD. SON-1210 at that dose was associated with a maximum serum concentration (C_max_) of 25 ng/mL, an area under the concentration-time curve (AUC_∞_) of 975 h*ng/mL and a terminal half-life (t_½_) of 18h.

The GLP toxicology study in cynomolgus monkeys formally evaluated the safety and tolerability of SON-1210 (**Table 2**). Repeated (days 1, 15, and 29) SC administration of SON-1210 at 15.6, 31.2, or 62.5 µg/kg/dose was well tolerated. Mild symptoms, consistent with rhIL-12 clinical effects in humans, occurred that were transient without off-target effects. The ‘no observed adverse effect level’ (NOAEL) was determined to be at least 62.50 µg/kg/dose of SON-1210, based on the overall results.

Clinical observations included transient effects, such as reduced appetite, hunched posture, and tremors, were transient and considered to be mildly adverse; no mortality related to SON-1210 was observed. SON-1210 had no effect on body weight, electrocardiographic, coagulation, or urinalysis parameters. Ophthalmic or macroscopic pathological findings were not observed. Hematology and clinical chemistry during the dosing period were indicative of affected hematopoiesis (bone marrow effect), increased red blood cell turnover, an inflammatory response, and mild dehydration. All observed findings returned to baseline during the recovery period by day 43. Following a 6-week recovery period, no SON-1210-related effects were noted, confirming the transient nature of the clinical effects of SON-1210.

Samples were collected for ADA analysis at various time points; only one of the monkeys had pre-existing IgG antibodies that recognized SON-1210. Following SON-1210 treatment, IgG ADA was detected in 14 of the 18 NHPs in the main group on day 15 and in all 18 by day 35 (**Table 3**) Sporadic IgM ADA was detected on days 15 and 35. Experience with administration of recombinant human cytokines to NHPs has led to the conclusion that the majority of these drugs, although inducing similar (if not identical) biologic effects, are highly immunogenic and can become neutralizing in these models (37).

**Table 3:** Anti-drug antibody (ADA) response in the NHP toxicology study.

		Pretest		Day 15		Day 35	
Dose group	Gender	IgG	IgM	IgG	IgM	IgG	IgM
**0 µg/kg**	**M**	–	–	–	–	–	–
	**F**	–	–	–	–	–	–
**15.62 µg/kg**	**M**	–	–	++	–	+++	–
	**F**	–	–	++	+	+++	–
**31.25 µg/kg**	**M**	–	–	++	–	+++	–
	**F**	+	–	++	+	+++	+
**62.50 µg/kg**	**M**	–	–	++	–	+++	–
	**F**	–	–	++	–	+++	–

NHPs were dosed with SON-1210 or placebo under GLP conditions on days 0, 15, and 29. Anti-drug antibodies (ADA) were assessed prestudy, at 15 days, and at sacrifice. ADA were analyzed as described and the highest response is shown.

-, absent; +, slight positive response; ++, definite positive response; +++, high positive response.

**Figure 6:**
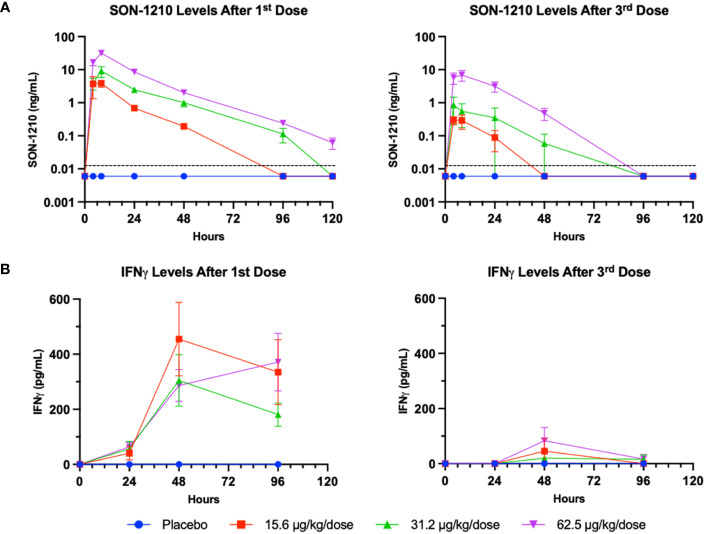
SON-1210 and IFNγ in NHP. Results from SON-1210 dosing after day 1 (left panels) and after day 29 (right panels). **(A)** A validated IL-12/IL-15 quantitative combination ELISA was used to ensure specificity. Drug levels are shown as the mean ± SEM for the SON-1210 dose response in NHP sera. The dotted black line represents the LLOQ; results below the level of quantitation are graphed as half the LLOQ by convention. **(B)** IFNγ levels were augmented after the 1^st^ dose at all dose levels and remained elevated for at least 96 h, although a significant dose-dependent response was not observed. After the 3^rd^ dose on day 29, a modest increase was still observed, despite the development of ADA impacting the effect of SON-1210 after the first dose.

The serum levels of SON-1210 (**Figure 6A**) were used to estimate multiple TK parameters using WinNonlin pharmacokinetic software following the first and third doses (on days 1 and 29, respectively) at specific time points 0-, 4-, 8-, 24-, 48-, 96-, and 120-h post dosing. As expected, a clear dose-dependent increase in both C_max_ and AUC_∞_ was evident overall (**Table 4**). The t_½_ values also increased as the dose increased, yielding nonlinear toxicokinetics that may be suggestive of target mediated drug disposition (TMDD) for SON-1210. The nonlinearity of the normalized values of C_max_/D and AUC_∞_/D also suggest TMDD. The variability in the data combined with the relatively small group sizes (two animals per sex per dose level) precluded the observation of any trends with respect to gender differences in the toxicokinetic data from the escalating dose studies. Three animals displayed similar concentration versus time profiles for the initial and third doses. However, the ADA titers for these animals were not significantly lower than the ADA titers of other subjects.

**Table 4:** Toxicokinetic data obtained from the NHP toxicology study.

Dose (µg)	Sex	Dose Day	Group	C_max_	C_max_/D	T_max_	T_last_	t_1/2_	AUC_∞_	AUC_∞_/D
				(ng/ml)	(kg*ng/ml/µg)	(h)	(h)	(h)	(h*ng/ml)	(h*kg*ng/ml/µg)
**17.1**	*M	1	2	3.9	0.229	8	48	10.0	64.8	3.8
**15.6**	F	1	2	6.1	0.392	4	48	8.5	79.6	5.1
**34.2**	M	1	3	12.5	0.364	8	96	18.4	267.1	7.8
**31.3**	*F	1	3	5.8	0.185	8	48	14.0	127.0	4.1
**68.4**	M	1	4	34.8	0.509	8	120	18.9	715.9	10.5
**62.5**	F	1	4	28.6	0.457	8	120	16.6	566.0	9.1
**15.6**	M	29	2	0.6	0.041	8	24	ND	ND	ND
**15.6**	F	29	2	0.4	0.023	4	8	ND	ND	ND
**31.3**	M	29	3	0.5	0.017	4	8	ND	ND	ND
**31.3**	*F	29	3	2.1	0.066	24	48	18.0	72.7	2.3
**62.5**	*M	29	4	6.2	0.099	8	48	13.4	143.4	2.3
**62.5**	F	29	4	8.0	0.128	4	48	12.1	215.7	3.4

Pharmacokinetic parameters calculated by noncompartmental analysis (NCA) of the mean concentration versus time profiles for subjects grouped by dose and sex for the initial dose (day 1) and third repeated dose (day 29). Intended dose levels were 15.625 µg/kg (Group 2), 31.25 µg/kg (Group 3), and 62.5 µg/kg (Group 4). Note that the initial dose on day 1 for all male subjects was 1.1x higher than the doses on day 15 and day 29 due to a calculation error. All parameters are estimates, as data was limited.

* Test subjects wherein the C_max_ value had to be incorporated as one of the three points in fitting the clearance phase to calculate the t_1/2_, AUC_∞_, and AUC_∞_/D, which is not standard practice, so the resultant values may be unreliable for these groups. ND, Not Determined; as there were less than three points in the clearance phase, even if the C_max_ was included.

SON-1210-related increases in IFNγ levels were observed at all doses within 24 to 48 h of SC administration (**Figure 6B**). However, IgG ADA appeared to suppress IFNγ responses following dosing on day 29. After the third dose on day 29 and by day 43, all cytokine levels, including plasma levels of IFNγ, IL-1β, IL-8, IL-6, IL-10, and TNFα, returned to baseline and did not suggest cytokine release syndrome. SON-1210 did not affect the absolute counts of total T lymphocytes, T helper cells, CTLs, B lymphocytes, or NK cell populations as assessed by FACS.

SON-1210 toxicity was compared to the reported toxicity of each recombinant human molecule. With SON-1210’s molecular weight of 105 kDa, the percentage contributions of IL-12 (60 kDa) and IL-15 (16 kDa) to the measured TK parameters of SON-1210 were 57% and 16%, respectively. The NOAEL in this study was 35.6 µg/kg for IL-12 and 10 µg/kg for IL-15. IL-12 concentration reached a maximum at 19.5 ng/mL and 4.6 ng/mL while the maximal IL-15 concentration was 4.6 and 1.3 ng/mL after the first and second administration, respectively. These levels were well-tolerated in monkeys, representing approximately 50 times the expected dose for safety and efficacy in human clinical trials.

## 4 Discussion

### 4.1 SON-1210 Mechanism of Action

The therapeutic-enhancing properties of the F_H_AB domain are rooted in its ability to bind albumin, the most abundant macromolecule in the blood (**Figure 1**). The F_H_AB platform demonstrated high affinity for both human and monkey albumin at the serum physiological pH of 7.2 and even more so at an acidic pH, which is often present in the TME. Single-chain IL-12 and native IL-15 were successfully linked in *cis* (6) to each side of the F_H_AB domain using (GGGGS)_5_ linkers (41), creating a novel bifunctional therapeutic drug candidate called SON-1210. The G4S linker construct is water soluble, non-immunogenic, flexible, and resistant to most proteases. The number of repeats used in the construct was optimized to avoid steric hindrance of the side arms. F_H_AB binds to albumin in the serum, which can target FcRn and GP60 receptors that are often upregulated in tumor endothelium to transport albumin into the TME. Tumors tend to utilize extracellular proteins as a source of amino acids to drive cellular growth (17), and the complex can bind tightly to SPARC in the acidic TME, enhancing retention and activation of local immune cells in solid tumors.

Given the complementary mechanisms of action of IL-12 and IL-15 and their cross-upregulation of receptors (2), various attempts have been made to evaluate their combined therapeutic effects in controlling the growth of tumors. For instance, SC injection of B78-H1 cells that were genetically modified to express IL-12 delayed melanoma growth in mice (42), an effect that was enhanced by IL-15 administration (43). IL-15 plays a role in preventing apoptosis of CD8^+^ memory cells and enhancing long-term memory surveillance. On the other hand, the use of albumin combined with therapeutic molecules has been shown to enhance transport to target sites, extend residence time in the TME, and improve efficacy while reducing toxicity (44–47). Various strategies, including fusing the therapeutic agent with HSA itself, native or recombinant, or with an albumin-binding domain, have improved the characteristics of pharmacological agents that target different mechanisms of action (48, 49). Given the potential of interleukins to be powerful therapeutic agents, along with their challenging pharmacokinetic and safety profiles, resolving some of these issues by interacting with native HSA is particularly important.

We recently began to study the properties of HSA to enhance the potential therapeutic benefits of IL-12 in ongoing clinical trials of the monofunctional IL12-F_H_AB molecule (SON-1010) (19, 20), which is distinct from other approaches being used to reduce its potential for toxicity (50). Alternative strategies have been employed to extend the pK of IL-12, such as linking it to an antibody (51), or to ensure a tumor effect by expressing IL-12 in oncolytic adenovirus for direct tumor injection (52, 53). Instead, we leveraged the properties of native HSA to target tumor tissue using the F_H_AB platform to extend the PK of IL-12 in humans, resulting in prolonged but controlled induction of IFNγ and decreased toxicity (19).

### 4.2 Characterization of the F_H_AB and SON-1210

To begin to characterize SON-1210, we sought to establish the best species to use as a model for further study and found that monkey albumin was closest to human albumin in its affinity to the F_H_AB domain using SPR, compared to rat, hamster, and canine albumin (**Figure 2**). The initial PK analysis of the F_H_AB platform was done in mice, comparing mIL12-F_H_AB or hIL15-F_H_AB to mIL-12 or hIL-15, respectively. The His-tagged versions of these molecules were produced to ensure that the native cytokines would not interfere with quantitation of each recombinant product. Extended PK of the SON-1210 molecule was subsequently confirmed in monkeys; further demonstration of the PK of the bifunctional molecule is planned for the first clinical study. SON-1210 promoted lymphocyte proliferation about the same as hIL-12 or hIL-15 *in vitro*. However, even though hIL12-F_H_AB (SON-1010) induced a similar amount of IFNγ release compared to hIL-12 *in vitro*, when both cytokines were present on the F_H_AB backbone substantially more IFNγ was produced by SON-1210, presumably due to the added IL-15 moiety that amplified the release. Finally, a biodistribution study of His-tagged mIL12-F_H_AB was done in mice that showed up to 3.1-fold increase in tumor accumulation compared to mIL-12 with prolonged tumor suppression.

SON-1210 was then studied using material manufactured for clinical use (**Figure 3**). Drug substance was produced using continuous perfusion after process development and qualification. The observed MW of 115 kDa by SDS-PAGE is consistent with appropriate glycosylation. Purity of > 97% was established by SE-HPLC. Charge heterogeneity is expected in biological products manufactured in CHO cells and can be demonstrated by iCIEF; SON-1210 showed mild variability, presumably due to glycosylation, and the results were within established specifications to proceed to further processing. Potency required evaluation of STAT4 activation by the IL-12 moiety, as well as STAT5 activation by the IL-15 moiety; HEK-Blue assays were used with specific cell lines to show SEAP induction in each case. Functional activity was established by detection of IFNγ release from human PBMCs. While macaque PBMCs were also able to release IFNγ, the quantity was expected to be less due to species differences in the native cytokines and receptors (37). Finally, the ability to stimulate T-cell proliferation was compared to the individual cytokines in the presence of antibody that blocked the complementing cytokine; SON-1210 outperformed both hIL-15 and hIL12-F_H_AB (SON-1010) used as controls.

Compared to the individual hIL-12 and hIL-15 interleukins, hIL12-F_H_AB-hIL15 showed similar proliferative activity *in vitro* (**Figure 2**). The ability to induce proliferation by each cytokine was also shown in isolation by blocking the complementary cytokine (**Figure 3**). When the cytokines were presented in *cis* and linked on the F_H_AB and studied using mIL12-F_H_AB-hIL15 *in vivo*, they showed greater tumor growth suppression and survival efficacy compared to the individual cytokines, even when those were used together (**Figure 4**). While three doses of the F_H_AB construct administered two days apart resulted in an even stronger suppression of tumor growth, this dosing strategy resulted in increased toxicity. As ADA could not have impacted the dose response in this timeframe, the closely spaced doses in the mice resulted in a marked induction of IFNγ, IL-10, and IL-12. This scenario is reminiscent of the severe toxicity observed in the first Phase 2 clinical study that started with repeated daily dosing of recombinant hIL-12, even though the original Phase 1 dose escalation study had been so successful when a ‘test dose’ had been given two weeks before daily dosing (54, 55). The difference between the safety results in the two rhIL-12 clinical studies can be ascribed to tachyphylaxis caused by induction of the suppressors of cytokine signaling (SOCS) (56), a class of cellular proteins that participate in negative feedback regulation of cytokine signaling. SOCS proteins appear to have allowed the toxic effects of IFNγ to be restrained in the Phase 1 study of rhIL-12. The lack of feedback and severe toxicity associated with immediate daily dosing in the Phase 2 study may have been similar to the aggravated toxicity in our B16F10 mouse tumor model, which we found when three doses of mIL12-F_H_AB-hIL15 were given every other day. If enough time is allowed before a second dose, the SOCS proteins can be induced to limit IFNγ toxicity.

Pharmacodynamic responses in the mice (**Figure 4**) and NHPs (**Figure 6**) showed moderate and prolonged increases in IFNγ levels with mild increases in other inflammatory cytokines, rapidly reversible cytopenia, and mild (except with 3x dosing in the mice) ALT elevation. FACS analysis in mice (**Figure 5**) showed a significant induction of activated T- and NK-cell responses, along with the conversion of M2 MDSCs to M1 APCs. Our *in vitro* data suggest that SON-1210 should stimulate the expected mechanisms associated with the therapeutic efficacy of the molecule, particularly T- and NK-cell stimulation and IFNγ production, which is important for local tumor surveillance (57). Such an effect has been previously reported using IL-15 attached to an albumin construct alone or in combination with a PD-L1 inhibitor (58, 59). Based on our FACS data, we conclude that treatment with mIL12-F_H_AB-hIL15 transformed the “cold” B16F10 tumor into a “hot” tumor. Interestingly, administration of equimolar doses of IL-12 and IL-15 failed to fully reproduce these effects in our model. These improved PD effects, compared to native interleukins, may be attributed to the F_H_AB platform’s tumor targeting by binding to albumin (17) and *cis* presentation of IL-12 and IL-15 (6), resulting in extended *in vivo* t_½_ and tumor retention for prolonged cytokine presentation to immune cells in the TME with limited toxicity.

### 4.3 Impact of Extended PK and Tumor Targeting by F_H_AB

The F_H_AB platform exhibits three pivotal characteristics *in vivo*: i) an enhanced t_½_ owing to the binding of albumin, ii) targeting of tumor tissue by binding to FcRn and GP60 receptors that are overexpressed in many solid tumors, and iii) retention in the acidic TME upon binding or rebinding to SPARC. Although we are currently focused on solid tumor indications, these findings suggest that the F_H_AB platform offers significant flexibility, allowing one or two therapeutic payloads for various modalities to benefit from t_½_ extension and/or HSA-based targeting of tumors and lymph nodes. While the current clinical product candidates have cytokines on either side, the F_H_AB platform can host various payloads, such as antibody motifs or other small molecules, as single- or bi-functional constructs, leveraging the F_H_AB’s on-off mechanism to gently interact with host tissues. The F_H_AB platform can bind, dissociate, and re-bind to albumin, setting up a dynamic equilibrium and slow elimination, allowing lower doses to be delivered and retained in the TME with greater effect in that space to enhance the therapeutic index. This is in contrast to drugs that use static covalent attachment to HSA or specific tumor targets, which typically do not re-bind, leaving the drug in its first location (45).

Our results suggest that by directing therapeutic cytokines to the TME using SON-1210, the anticipated therapeutic efficacy based on their biological activity can be achieved. In addition, our approach addresses the paramount safety and tolerability factors, which have traditionally hindered the use of therapeutic cytokines in the treatment of solid tumors, by improving the therapeutic index. The candidate drug activates the immune response in the TME, which upregulates IFNγ and increases PD-L1 expression, potentially making checkpoint inhibitors more active (25). It can also be combined with cell-based therapy to extend the half-life and activity of CAR-T cells (60). This novel approach could help to redefine the cancer battle and the results presented here position SON-1210 for its initial human cancer trials.

## Data availability statement

The raw data supporting the conclusions of this article will be made available by the authors, without undue reservation.

## Ethics statement

Ethical approval was not required for the studies on humans in accordance with the local legislation and institutional requirements because only commercially available established cell lines were used. The animal studies were approved by Institutional Animal Care and Use Committee (IACUC) of Invivotek and the IACUC of Charles River Laboratories. The studies were conducted in accordance with the local legislation and institutional requirements.

## Author contributions

JC: Conceptualization, Data curation, Formal Analysis, Methodology, Project administration, Supervision, Writing – original draft, Writing – review & editing, Software. SD: Writing – original draft, Writing – review & editing, Conceptualization, Data curation, Project administration, Supervision. DR: Conceptualization, Data curation, Formal Analysis, Writing – original draft, Writing – review & editing. SM: Conceptualization, Formal Analysis, Project administration, Supervision, Writing – original draft, Writing – review & editing. GH: Data curation, Formal Analysis, Visualization, Writing – original draft, Writing – review & editing. RB: Conceptualization, Data curation, Formal Analysis, Investigation, Methodology, Supervision, Writing – review & editing. RE: Data curation, Formal Analysis, Investigation, Methodology, Software, Supervision, Validation, Visualization, Writing – review & editing. RK: Conceptualization, Data curation, Formal Analysis, Visualization, Writing – original draft, Writing – review & editing, Software, Validation. PM: Conceptualization, Funding acquisition, Project administration, Supervision, Writing – original draft, Writing – review & editing.
