# Supplementary material for: SON-1210 - a novel bifunctional IL-12 / IL-15 fusion protein that improves cytokine half-life, targets tumors, and enhances therapeutic efficacy
Source: Front Immunol. 2023 Dec 20;14:1326927. doi: 10.3389/fimmu.2023.1326927 (PMC10798159; doi:10.3389/fimmu.2023.1326927)
Supplement: Supplementary file 1 [file DataSheet_1.pdf]

Supplementary Material

1 SON-1210 extra in vitro method details

1.1 Albumin Binding of SON-1210 by Surface Plasmon Resonance (SPR)

Five individual Biacore™ SPR CM5 sensor chips (Cytiva Cat# 29104988) were chemically conjugated to the albumins from the five species described. The kinetic constants were measured at both pH 5.8 and pH 7.4 to determine whether IL12-F_HAB-IL15 binds albumin at neutral pH in serum and then remains attached at the lower pH of the tumor microenvironment (TME) after uptake in that space. Approximately 300 resonance units (RUs) of human serum albumin (HSA), cynomolgus monkey serum albumin (MSA), rat serum albumin (RSA), and about 1000 RU of canine serum albumin (CSA) and Syrian hamster serum albumin (HamSA), were immobilized onto CM5 sensor chips using the Biacore target ligand immobilization program. An extra flow cell went through the immobilization procedure in the absence of albumin and was used for reference subtraction. hIL12-F_HAB-hIL15 (SON-1210) was buffer exchanged into running buffer (PBS buffer with 0.05% Tween-20, pH 5.8 or 7.4) and 2-fold serially diluted from 1600 nM to 25 nM. Each dilution was injected onto an albumin coated chip for 100 seconds at a flow rate of 30 µL/min and the dissociation phase was monitored for 200 seconds, followed by a 1 min regeneration pulse using 3.85 M $MgCl_2$ at a flow rate of 10 µL/min. Association and dissociation rates of the reference- and blank-subtracted sensorgrams were calculated using the two-state reaction model fit analysis (Biacore Evaluation Software).

Figure S1: Binding of SON-1210 to Serum Albumin from Different Species

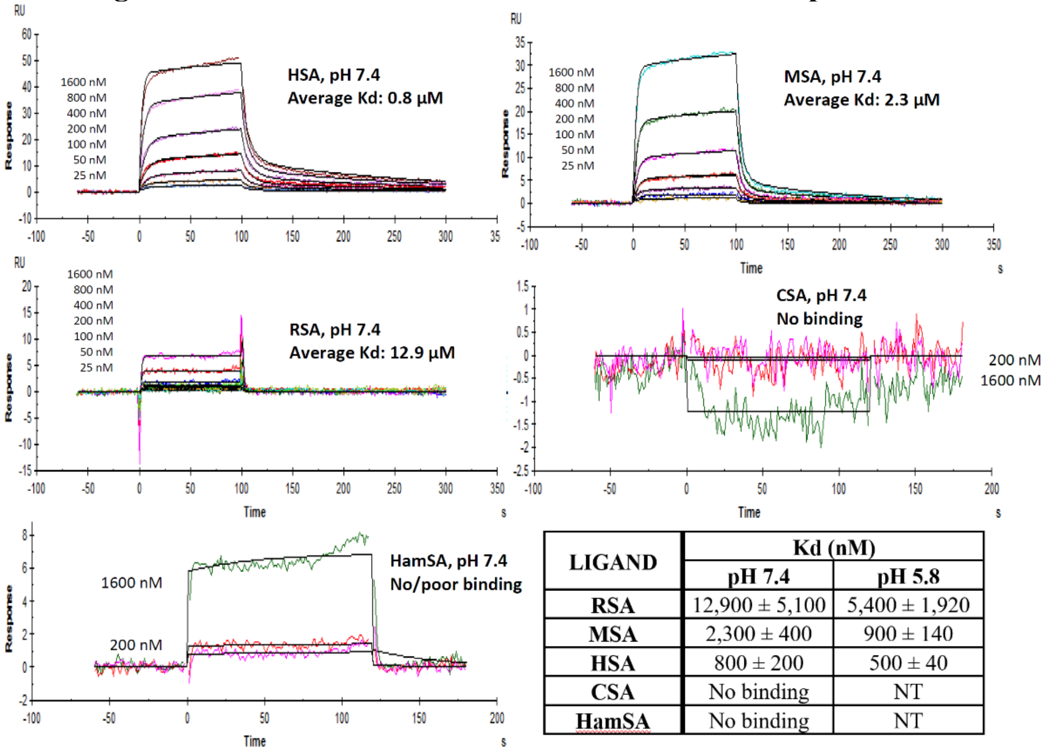

LIGAND	Kd (nM)	
	pH 7.4	pH 5.8
RSA	12,900 ± 5,100	5,400 ± 1,920
MSA	2,300 ± 400	900 ± 140
HSA	800 ± 200	500 ± 40
CSA	No binding	NT
HamSA	No binding	NT

Surface plasmon resonance curves at pH 7.4 of species-specific serum albumin binding to SON-1210. The K_d is the dissociation constant; a lower K_d indicates stronger binding. Abbreviations: RSA = rat serum albumin, MSA = monkey serum albumin, HSA = human serum albumin, CSA = canine serum albumin, HamSA = hamster serum albumin.

1.2 F_HAB Binding to Human or Macaque Serum Albumin in Solution

Solution K_d values were determined by a modification of the method of Friguet et al. (32). ELISA plates were coated for 1 hour at with either 6 µg/mL HSA or 6 µg/mL MSA in pH 7.4 PBS, washed three times with pH 7.4 PBS + 0.05% Tween 20, and blocked for 1 hour with either pH 7.4 PBS + 0.25% Tween 20 or pH 6.2 PBS + 0.25% Tween 20. All other steps in the procedure were performed in PBS buffer + 0.05% Tween 20 at the same pH as the block step. While the ELISA plates were being coated, samples that contained 2 nM SON-1210 were incubated with different concentrations of either HSA or MSA in either pH 7.4 PBS + 0.05% Tween 20 or pH 6.2 PBS + 0.05% Tween 20. At each albumin concentration, samples were prepared in triplicate or quadruplicate. On the same plate, SON-1210 dilutions were incubated in the same buffer with no albumin as a standard curve. After 1 hour, 0.05 mL aliquots of each sample were transferred to the coated and blocked ELISA plate, incubated one hour, washed three times, and then incubated for one hour with an anti-Human IL-12p70 biotinylated detection antibody (ThermoFisher CUST77216, 125 X dilution). After three washes, the samples were incubated for 1 hour with 0.5 µg/mL Streptavidin-HRP (ThermoFisher 21130), washed four times, and visualized with TMB (Sera Care 5120-0083) for 10 minutes before quenching with 0.05 mL 1 M HCl and reading the plate at 450 nm.

Unlike the usual K_d calculations, where the ligand:receptor complex is expressed as a fraction of total receptor, in these experiments the actual concentration of the SON-1210:albumin complex can be determined. This is done by subtracting concentration of unbound SON-1210, determined from the assay response and the standard curve run on the same plate, from the total SON-1210 added to each well. This allows a K_d value to be determined at each concentration of added albumin. The K_d values for each experiment were calculated by first averaging the sample responses for each albumin concentration and then determining the concentration of unbound SON-1210 using the SON-1210 standard curve on each plate. Since the concentrations of unbound SON-1210, total SON-1210, and total albumin are then known, the K_d at each albumin concentration can be calculated:

$$K_d = \frac{(\text{SON-1210})(\text{Albumin})}{(\text{SON-1210:Albumin})} = \frac{(\text{SON-1210})(\text{Albumin}_{\text{Total}} - \text{SON-1210:Albumin})}{(\text{SON-1210}_{\text{Total}} - \text{SON-1210})}$$

The K_d values from the linear portion of response vs log(albumin) curves were averaged to obtain the reported K_d.

1.3 SPARC Binding to Human Serum Albumin

Solution K_d values were determined by a modification of the method of Friguet et al. (32). ELISA plates were coated with HSA as above. While the ELISA plates were being coated, samples that contained 0.15 nM Biotin (B)-SPARC (Sinobiological Cat# 10929-H08H) were incubated in solution in polypropylene microtiter plates with different concentrations of HSA (3000 nM to 3 nM in 2 fold dilutions) in pH 6.0 PBS + 0.05% Tween 20. At each B-SPARC:HSA concentration, samples were prepared in triplicate. On the same plates, B-SPARC dilutions (0.15 nM to 0.0002 nM; 2 fold dilutions) were incubated in the same buffer with no HSA as a standard curve. After 1 hour, 0.05 mL aliquots of each SPARC:HSA and SPARC sample were transferred to the coated and blocked HSA ELISA plate, incubated for one hour, washed three times, and then incubated for one hour with 0.25 µg/mL Streptavidin-HRP (ThermoFisher). The wells were then washed four times and visualized with TMB (Sera Care) for 20 minutes before quenching with 0.05 mL 1 M HCl and reading the plate at 450 nm.

In these experiments, the actual concentration of the B-SPARC:HSA complex was determined. This is done by subtracting the concentration of unbound B-SPARC, determined from the assay response and the standard curve run on the same plate, from the total B-SPARC added to each well. This allows a K_d value to be determined at each concentration of added albumin. The K_d values for each experiment were calculated by first averaging the triplicate sample responses for each B-SPARC:HSA concentration and then determining the concentration of unbound B-SPARC using the B-SPARC standard curve on each plate. Since the concentrations of unbound B-SPARC, total B-SPARC, and total HSA are then known, the K_d at each HSA concentration can be calculated:

$$K_d = \frac{(\text{B-SPARC})(\text{HSA})}{(\text{B-SPARC:HSA})} = \frac{(\text{B-SPARC})(\text{HSA}_{\text{Total}} - \text{B-SPARC:HSA})}{(\text{B-SPARC}_{\text{Total}} - \text{B-SPARC})}$$

1.4 Retention of the F_HAB in Tumor Tissue

An early proof-of-concept study was conducted (13) to show tumor retention of the F_HAB scFv using the mouse 4T1 model, which expresses TGFβ. Once the tumors had grown to 100 mm^3, the mice were injected with 100 µg IV of His-tagged F_HAB, an anti-TGFβ scFv, or anti-TGFβ-F_HAB per mouse, then the tumors were harvested after 0.5- to 24-hours (Figure S2). Western blot analysis using a His-tag detection system showed that the F_HAB was present at 0.5-hours, its level peaked at 4-hours, and it was still detectable through 24-hours. The anti-TGFβ could also be detected at 0.5-hours but declined at 4-hours and was undetectable at 12- and 24-hours. The anti-TGFβ was linked to the F_HAB, creating a 50 kD anti-TGFβ-F_HAB molecule, which was shown to be present in the tumors at 0.5 hours and was still detectable through 24 hours. Thus, the F_HAB itself accumulated in the tumor, as well as a F_HAB that had been linked to another macromolecule.

Figure S2: FHAB Uptake and Retention in Tumor Tissue

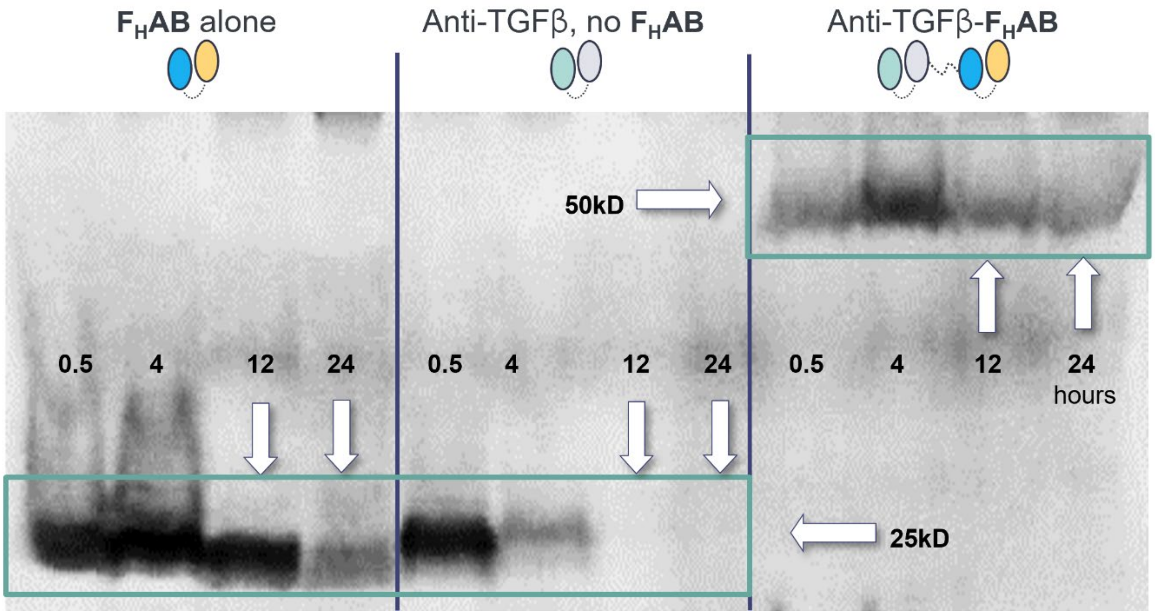

Western blot analysis of tumor accumulation of the F_HAB, anti-TGFβ, or anti-TGFβ-F_HAB at 0.5- to 24-hours after IV injection.

1.5 Culture of Human and Macaque PBMCs

Human (IQ Biosciences) and cynomolgus monkey peripheral blood mononuclear cells (PBMCs, IQ Biosciences) were thawed and cultured at 37°C in 5% CO_2 humidified air in RPMI 1640 complete medium (ATCC) supplemented with 10% heat-inactivated Fetal Bovine Serum (FBS, Gibco), 2 mM L-glutamine (ATCC), and 1% penicillin-streptomycin solution (ATCC).

1.6 Potency of SON-1210 (Figure 3B)

Both human IL-12 and IL-15 act on PBMCs, stimulating STAT4 and STAT5 phosphorylation, respectively, producing interferon gamma (IFNγ) and enabling proliferation of T-cells. Recombinant human IL-12 (Cat# 200-12), human IL-15 (Cat# 200-15), human IL-2 (Cat# 200-02), and human IL-8 (Cat# 200-08) were purchased from Peprotech. The HEK-Blue IL-2 reporter cell kit (Cat# hkb-il2) and the HEK-Blue IL-12 reporter cell kit (Cat# hkb-il12) were purchased from InvivoGen. To determine the potency of the IL-12 component of SON-1210, HEK-Blue IL-12 cells were seeded onto a 96-well plate at approximately 50,000 cells/well in media containing 10% FBS and treated with SON-1210, hIL-12 (positive control), hIL-15 (negative control), and hIL-8 (negative control). After incubating the plates at 37 °C for 20 to 24 hours, 20 µL of each induced HEK-Blue IL-12 cell supernatant was added to a 96-well plate and mixed with 180 µL Quanti-Blue solution. The STAT4 response was determined by measuring SEAP concentrations in supernatants that were detected and quantified via absorbance readings at 620 nm after incubating the plates for 4 hours. The results of two experiments were averaged.

The hIL-2 and hIL-15 cytokines are closely related, and both belong to the IL-2 family. They share the heterodimeric CD122 (IL-2Rβ)/CD132 (IL-2Rγ) receptor to deliver their signals into target cells. To determine the potency of the IL-15 component of SON-1210, HEK-Blue IL-2 reporter cells express a STAT5-inducible secreted embryonic alkaline phosphatase (SEAP) reporter gene that is triggered upon binding of hIL-15 to the hIL-2 receptor. HEK-Blue IL-2 cells were seeded onto a 96 well plate at approximately 50,000 cells/well in media containing 10% FBS and exposed in separate wells to SON-1210, hIL-15 (positive control), hIL-12 (negative control), and hIL-8 (negative control). After incubating the plates at 37 °C for 20 to 24 hours, 20 µL of each induced HEK-Blue IL-2 cell supernatant was added to a 96 well plate and mixed with 180 µL Quanti-Blue solution. The STAT5 response was determined by measuring SEAP concentrations in the supernatants that were detected and quantified via absorbance readings at 620 nm after incubating the plates for 4 hours. The results of two experiments were averaged.

1.7 Production of IFNγ (Figure 3C)

PBMCs were stimulated with 4 µg/mL of PHA-L (ThermoFisher) for 72 h followed by 10 IU/mL IL-2 (Peprotech) incubation for 24 h. Subsequently, the cell concentration was adjusted to 1 x 10^5 cells/mL and 100 µL aliquots were added to the wells of a 96-well plate (1 x 10^4 cells/well) in media supplemented with 10% FBS. Cells were then incubated with SON-1210, hIL-12, or media alone for another 48 h. Plates were then centrifuged at 150 g for 10 minutes and the supernatant was removed for analysis. The IFNγ levels of the supernatants were determined by species-specific ELISA kits (Human IFNγ High Sensitivity ELISA Kit, Abcam; Monkey IFNγ ELISA PRO Kit, Mabtech) according to the manufacturers' instructions. Results are represented as mean ± SEM of duplicate wells from a single experiment.

1.8 Stimulation of T- and NK-cell proliferation (Figure 3D)

The ability of the IL-12 component of SON-1210 to promote cell proliferation was studied in human PBMCs depleted from monocytes (to avoid intrinsic IL-12 interference) and an anti-IL-15 antibody was used to block IL-15 stimulation using the CellTiter Aqueous One Solution Cell Proliferation kit (Promega Cat# G3580), which uses media supplemented with 5% FBS. The cells were stimulated with PHA-L at 4 µg/mL and allowed to proliferate for 3 days. After 3 days, human IL-2 (50 IU/mL) was added to the culture. After 24 h, the media containing human IL-2 was removed and cells were incubated with media containing SON-1210 or IL-15 (with or without anti-IL-15 antibody, R&D Systems). Different concentrations of hIL-15 (PeproTech, Cat# 200-15) or SON-1210 (0.016-50 pM) were incubated with the fixed concentration of anti-IL-15 antibody (50 µg/mL) at 24°C for 30 mins before adding the mixtures to the PHA-L and IL-2 pre-treated human PBMCs for 48 hours. At the end of incubation, cell proliferation was determined by adding 100 µL of Cell Titer-Glo luminescent reagent (Promega) and the luminescence for each well was recorded. To study the IL-15 component of SON-1210, the same procedure was used except that IL12-F_HAB was used instead of IL-15 as a control and anti-IL-12p70 antibody (25 µg/mL) was used to block the IL12-F_HAB.

2 In vivo efficacy in tumor-bearing mice

Eight- to nine-week-old female C57BL/6 mice were received from Jackson Labs West and group housed on a 12-hours dark/light cycle for 1 to 3 weeks in a humidity- and temperature-controlled facility before entering the experiment. They were given *ad libitum* access to food and water during the entire experiment. After inoculation, the animals were checked daily for morbidity and mortality and any effects of tumor growth on mobility, food and water consumption, body weight gain/loss, eye/hair matting and any other abnormal effects. Observed clinical signs or death were recorded. Animals in continuing deteriorating condition were observed for recovery or were euthanized.

B16F10 murine melanoma cells were maintained as a monolayer culture in DMEM media supplemented with L-glutamine (2 mM), glucose (4.5 g/L), sodium pyruvate and 10% heat-inactivated fetal calf serum at 37°C in the presence of 5% CO2. The tumor cells were routinely subcultured twice weekly and were cultured for 2 to 3 passages (P). The cells in an exponential growth phase were harvested and counted for tumor inoculation at P7.

Mice received a subcutaneous inoculation in right lower flank with a single cell suspension of 0.2×10^6 cells in 0.1 ml of serum free, phenol free DMEM. Grouping and treatments were initiated when the mean tumor volume reached approximately 90 to 100 mm^3, seven days after inoculation, defining Day 0 of the study. Any tumors that grew intradermally, intramuscularly or were irregularly shaped (W- or U-shaped) were excluded from the study. Tumor-bearing mice were assigned to treatment groups using a computer-generated randomization procedure. All groups were further subdivided for bleeding at days 0, 3 or 8, FACS analysis or efficacy evaluation. The Tumor-Bearing Placebo group received the melanoma cells and placebo administration while the Non-Tumor Bearing (naïve) group received no treatment and was used for hematology and clinical chemistries on Day 0. Treatments were administered by IV injection in 200 µL of 0.02% Tween 20 in PBS on the day of dosing, Day 0.

Body weights and tumors were measured three times per week until reaching the IACUC-approved animal protocol endpoint (tumor volume of 1800 mm^3), at which point the animals were euthanized. Tumors were measured in two dimensions using calipers. Tumor volumes were calculated using the formula: Volume (mm^3) = (length × width^2)/2.

Blood was collected via the retro-orbital vein under isoflurane anesthesia and collected in EDTA-coated tubes. Hematological analysis of the peripheral blood was performed on the Hemavet analyzer (Drew Scientific) using the mouse setting. The number of total white blood cells (WBC), lymphocytes (LY), eosinophils (EO), neutrophils (NE), monocytes/macrophages (MO), basophils (BA), red blood cells (RBC) and platelets (PLT) were determined. RBCs and platelet counts are shown in Figure S3.

Figure S3: Red Blood Cells and Platelets from Days 3 and 8

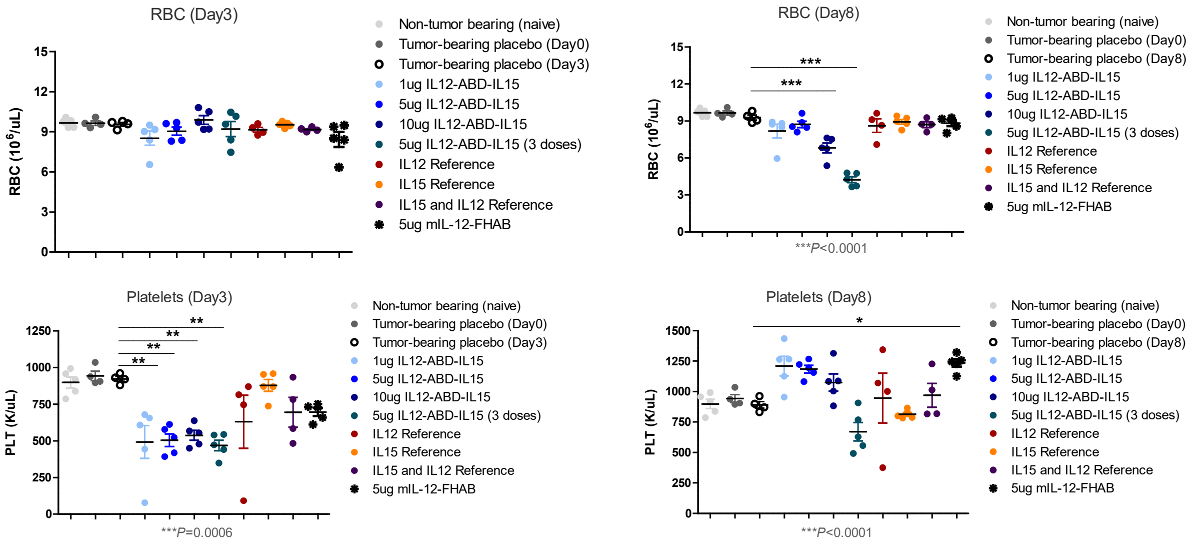

Scatter plots are shown for RBCs and platelets in each group on the indicated sampling day after a single dose on day 0, or three doses in the second 5 µg group on days 0, 2, and 4.

Blood samples of Days 3 and 8 were used for serum preparation after coagulation for 30 minutes at room temperature and centrifugation at 1000g for 15 minutes at 4°C. 1:1 diluted serum (PBS) was analyzed with a clinical chemistry analyzer (Alfa Wassermann Vet Axcel) to detect alanine aminotransferase (ALT), aspartate amino transferase (AST), creatinine (Creat), and total bilirubin (TBIL).

Tumors samples were collected when the average tumor size was ~250 mm^3 on Day 3. Single cell suspensions from freshly collected tumors were prepared by transferring individual tissues into gentleMACS C Tubes containing 5 ml of RPMI and placed onto gentle tissue dissociator (MACS, Miltenyi Biotec). Single cell suspensions were filtered through Falcon 100 µm nylon filters and centrifuged. The staining panel included markers for live/dead, CD45, TCR-beta, CD8, CD4, CD25, FoxP3, IFNγ, CD49b, F4/80, CD206, CD11b, CD11c and MCHII. The gating strategy is listed in Table S1. The study design is shown in Figure S4, with graphic tumor results in Figure S5 and graphic spleen results in Figure S6.

Figure S4: Study Design for FACS Analysis of B16-F10 Tumors and Spleens

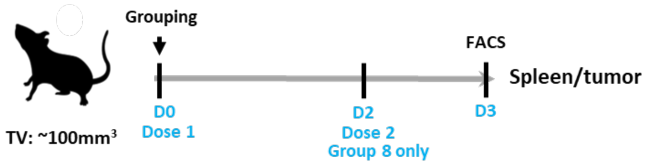

Groups	Treatment
Group 1	Tumor Bearing Placebo
Group 3	5 µg mIL12-F$_H$AB-hIL15
Group 7	IL15-Reference & IL12-Reference
Group 8	5 µg mIL12-F$_H$AB-hIL15 (2 doses)
Group 9	mIL12-F$_H$AB

FACS analysis:

1. 4x10^6 cells /per sample
2. 5hr stimulation (1.5hr/3.5hr)
3. ON in Transcriptional factor perm/fix buffer

Female C57BL/6 mice received a subcutaneous inoculation of 0.2×10^6 B16F10 cells. Grouping and treatments were initiated when the mean tumor volume reached approximately 90 to 100 mm^3, seven days after inoculation. Animals from all groups were dosed once via an IV injection into the tail vein on day 0 following grouping, with the exception of Group 8, which received two doses.

Table S1: FACS Gating Strategy for Tumor and Spleen Samples

Gating strategy	Defined cell population
BV510 Ghost$^-$	Live cells
Live/CD45$^+$	Immune cells
Live/CD45$^+$TCR$^+$CD49b$^-$	T cells
Live/CD45$^+$TCR$^+$CD49b$^-$CD4$^+$	CD4$^+$ T cells
Live/CD45$^+$TCR$^+$CD49b$^-$CD4$^+$IFNγ	Th1 cells
Live/CD45$^+$TCR$^+$CD49b$^-$CD4$^+$CD25$^+$FoxP3$^+$	Regulatory T (Treg) cells
Live/CD45$^+$TCR$^+$CD49b$^-$CD8$^+$	CD8$^+$ T cells
Live/CD45$^+$TCR$^+$CD49b$^-$CD8$^+$IFNγ$^+$	Cytotoxic CD8$^+$ T cells
Live/CD45$^+$TCR$^-$CD49b$^+$	NK cells
Live/CD45$^+$TCR$^-$CD49$^-$CD11b$^+$	Myeloid cells
Live/CD45$^+$TCR$^-$CD49$^-$CD11b$^+$F4/80$^+$	Macrophages
Live/CD45$^+$TCR$^-$CD11b$^+$F4/80$^+$MHCII$^+$CD206$^-$	M1 macrophages
Live/CD45$^+$TCR$^-$CD11b$^+$F4/80$^+$MHCII$^-$CD206$^+$	M2 macrophages
Live/CD45$^+$TCR$^-$CD49$^-$CD11c$^+$	Dendritic cells (DCs)

Figure S5: FACS Gating Strategy and Populations in B16-F10 Tumors on Day 3

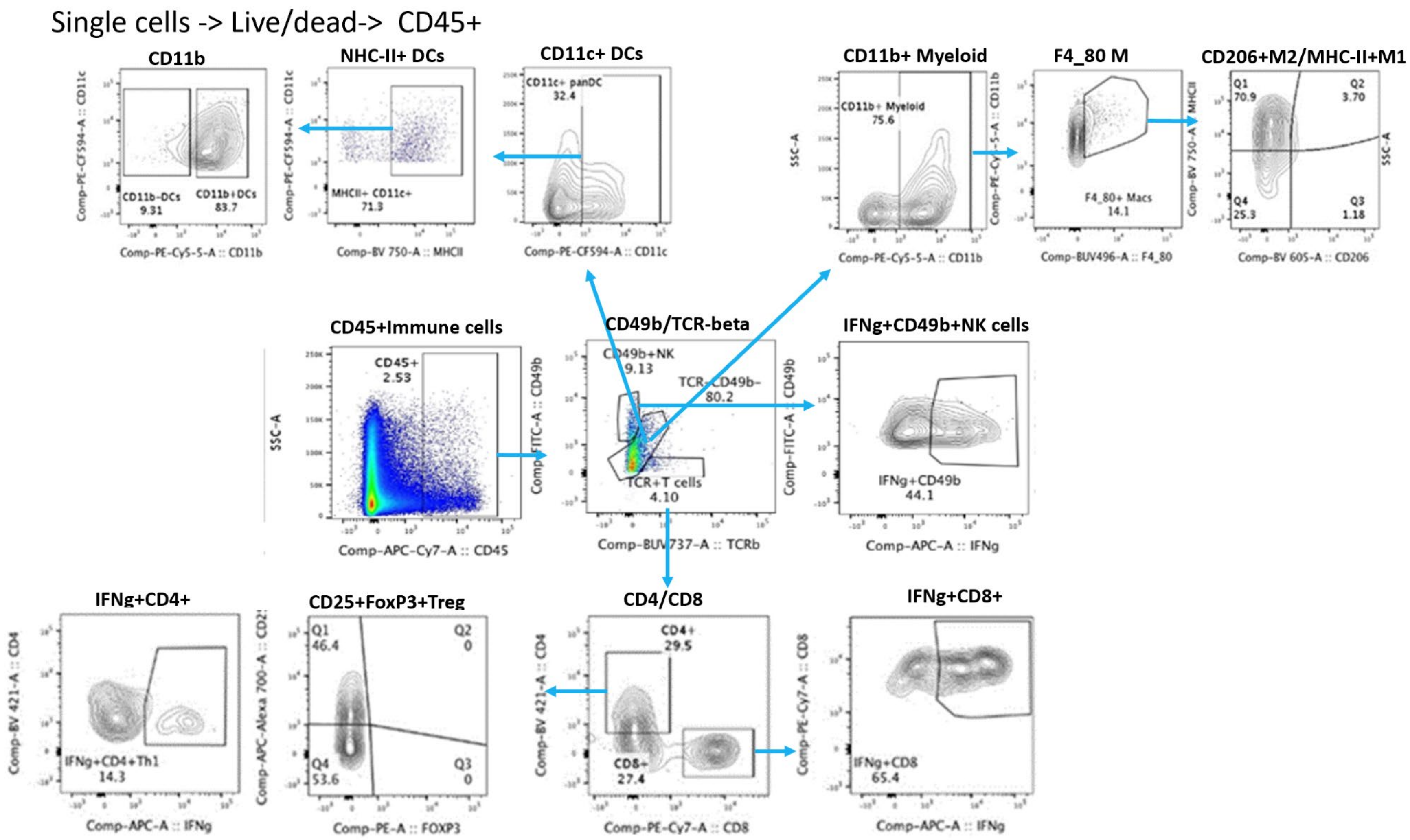

Tumors samples were collected when the average tumor size was ~250 mm3 on Day 3. The staining panel included markers for live/dead, CD45, TCR-beta, CD8, CD4, CD25, FoxP3, IFN-γ, CD49b, F4/80, CD206, CD11b, CD11c and MCHII (Table S1).

Figure S6: FACS Gating Strategy and Populations in B16-F10 Spleens on Day 3

A: Spleen Gating Results

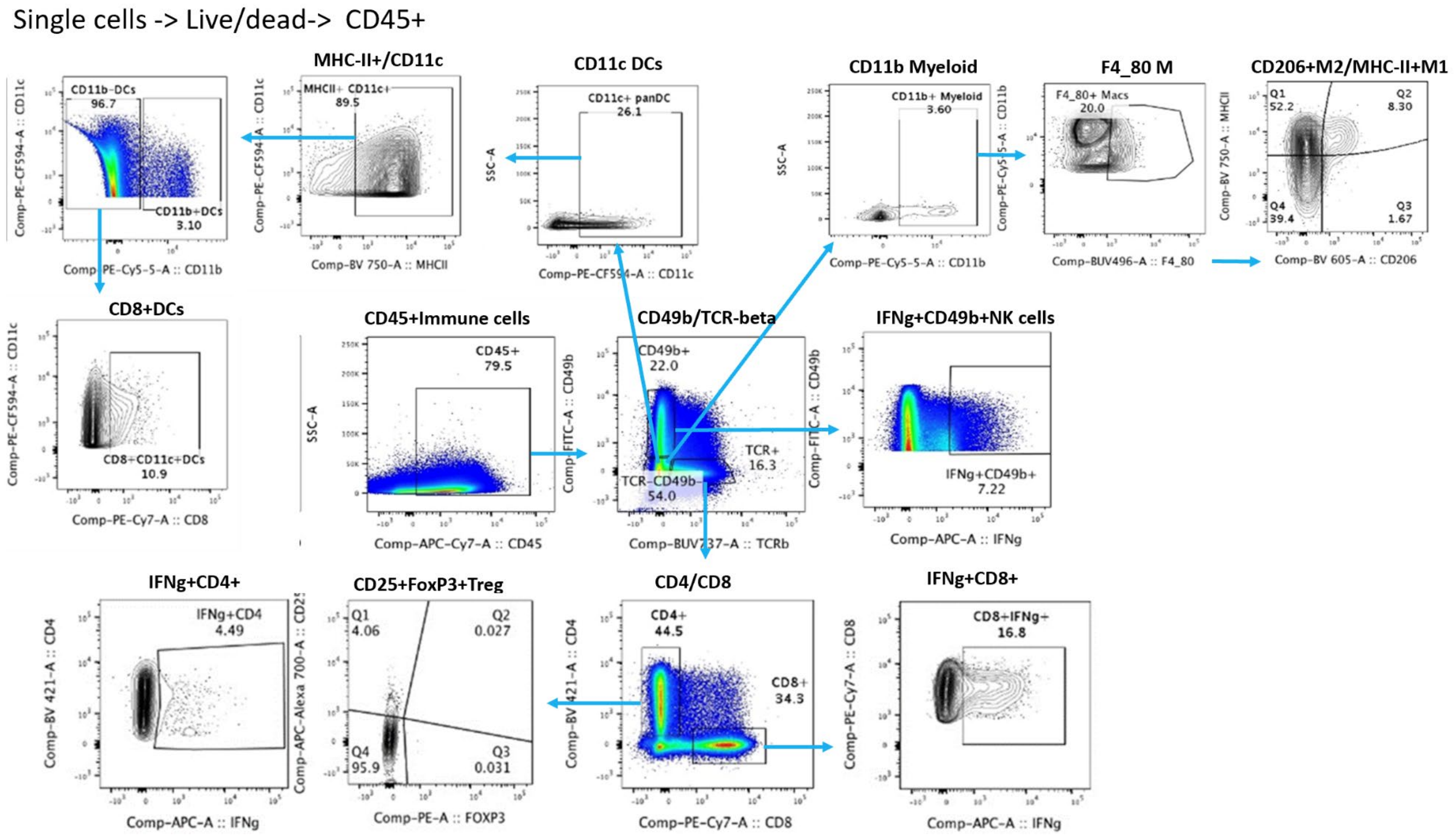

B: Spleen NK and T-cell Populations

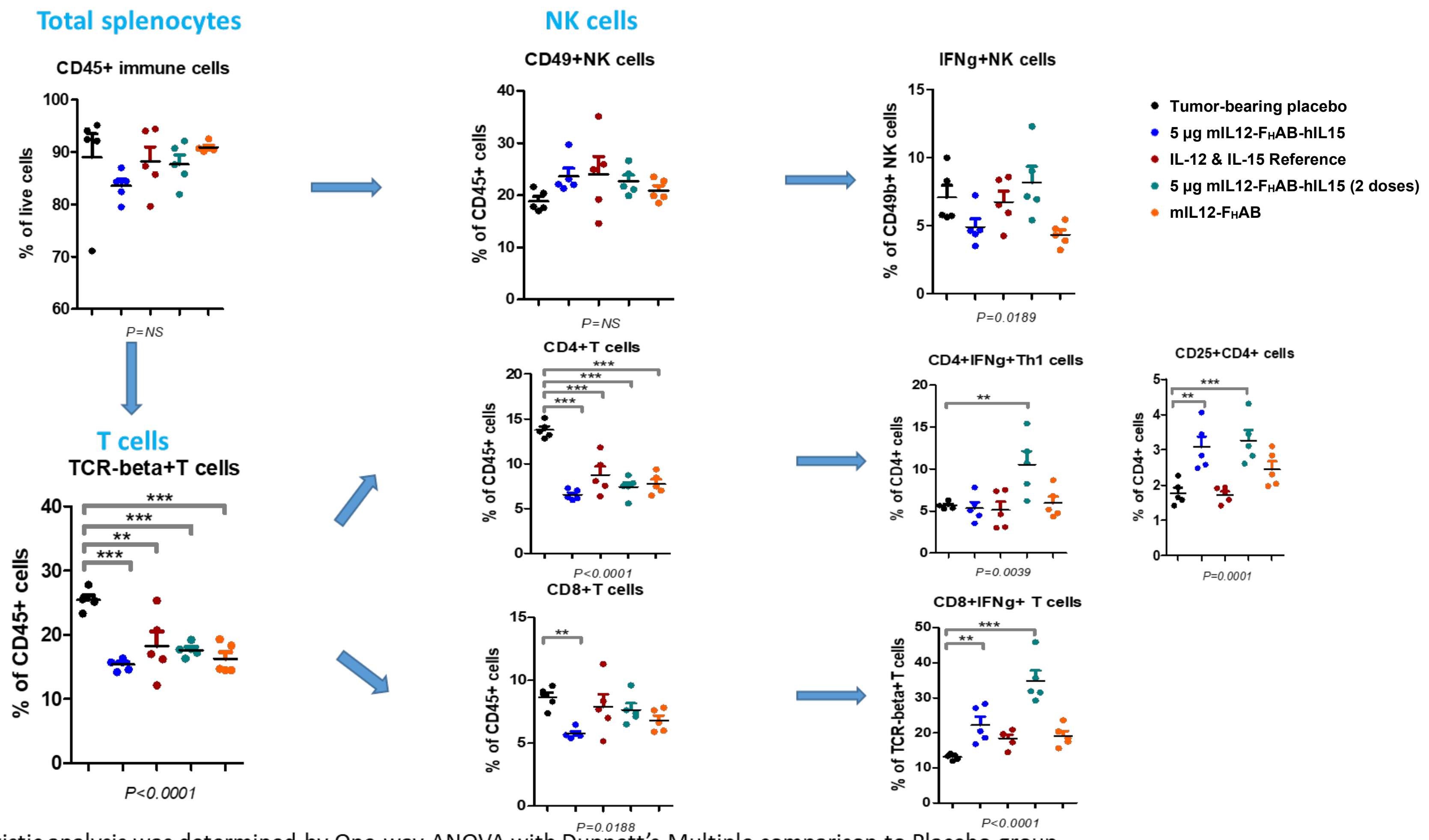

Statistic analysis was determined by One-way ANOVA with Dunnett's Multiple comparison to Placebo group

C: Spleen Myeloid Populations

CD11b+ total Myeloid cells

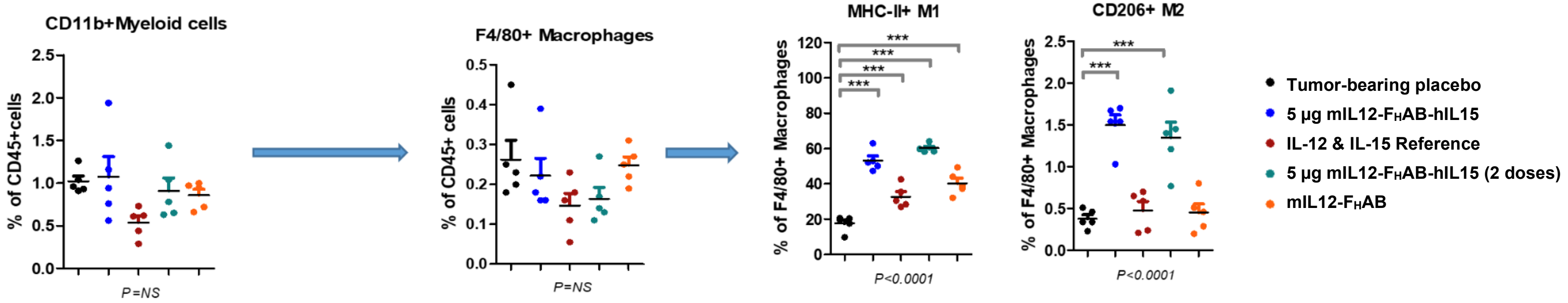

CD11c+ total dendritic cells

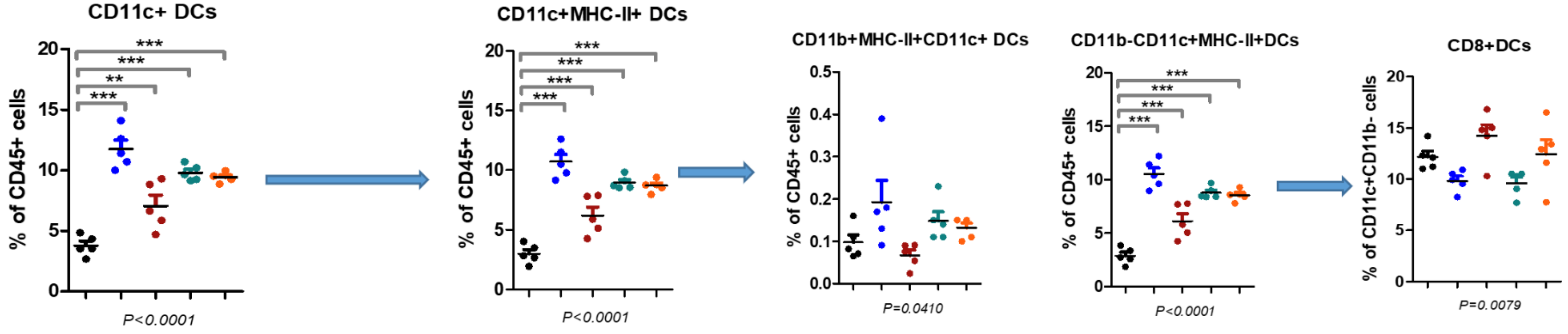

Statistic analysis was determined by One-way ANOVA with Dunnett's Multiple comparison to Placebo group

D: Spleen Treg Population

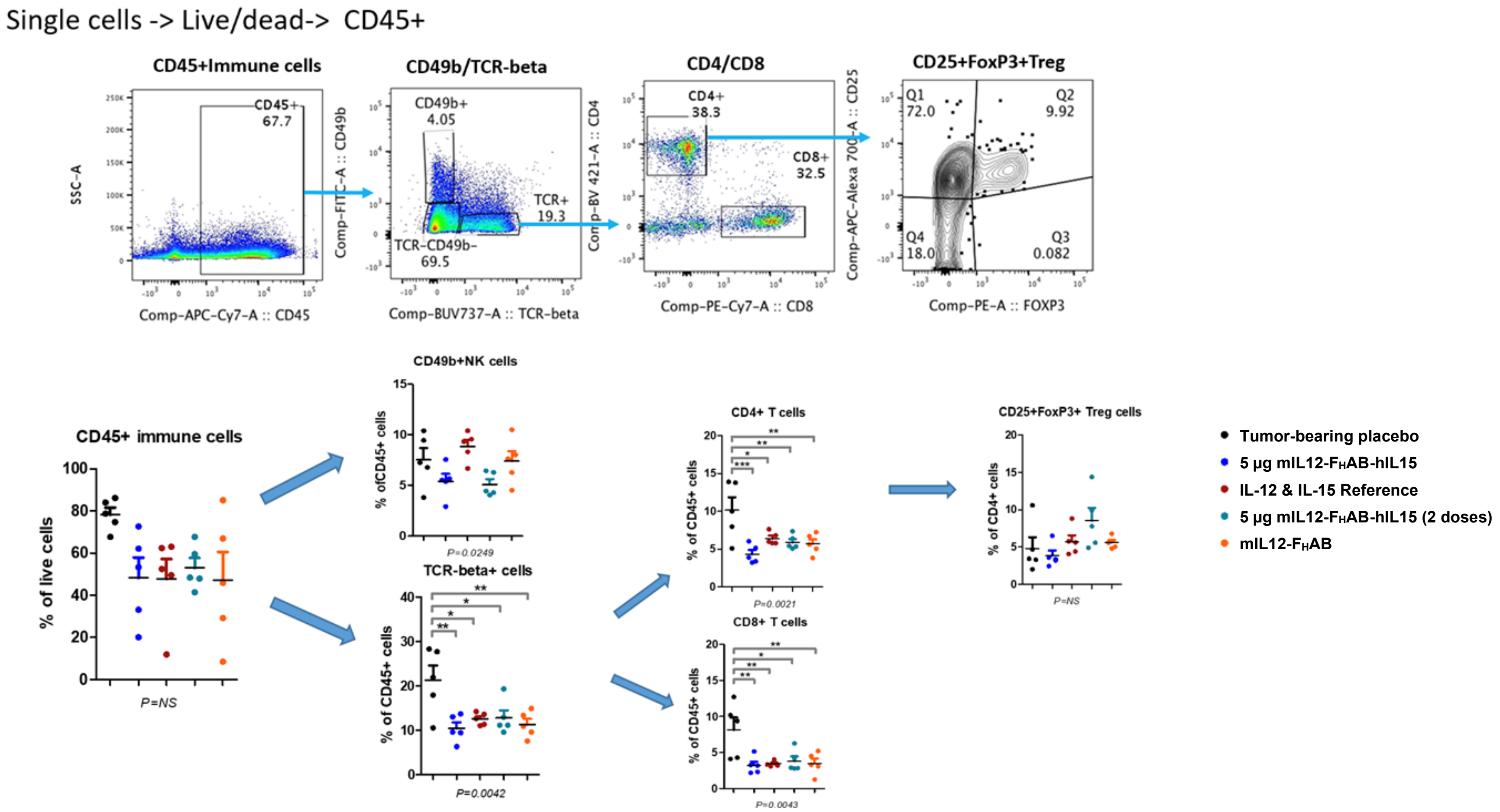

Spleen samples were collected when the average tumor size was ~250 mm3 on Day 3. The staining panel included markers for live/dead, CD45, TCR-beta, CD8, CD4, CD25, FoxP3, IFN-γ, CD49b, F4/80, CD206, CD11b, CD11c and MCHII (Table S1). (**A**) NK and T-cell gating results; (**B**) NK and T-cell populations by group; (**C**) Myeloid cell populations by group; (**D**) Derivation of Treg gating and Treg population by group.

3 GLP evaluation of potential toxicological effects of SON-1210 and its toxicokinetic effects

Safety, toxicology and the toxicokinetic effects of SON-1210 were evaluated in males and females cynomolgus monkeys (2 to 4 years of age, 2.5 to 6 kg at study initiation) using subcutaneous repeat bolus administration on experimental days 1, 15 and 29. Dose range was determined from a previous non-GLP experiment (data not shown). The present GLP toxicology study design is shown in Tables 2 and 3 for males and females, respectively. Briefly 4 dose groups were set for both males and females. Males and females received a constant dose at each administration (vehicle, 15.62, 31.25 and 62.59 µg/kg/dose). Each dose group for both genders was composed of 3 animals. In addition, recovery groups of 2 animals were established for the vehicle- and highest dose-groups. Animals were monitored for mortality (at least twice daily), clinical observations (weekly), body weight (weekly), food consumption (daily). Ophthalmologic monitoring was performed using fundoscopy and biomicroscopic examination under 1% tropicamide and Ketamine at pre-study and week 2 and 4 and last recovery week. Electro-cardiology was evaluated with qualitatively exam of the waveforms and quantitative measurement of HR and/or RR interval, PR and QT intervals, calculation of QTc (using Van de Water's formular) as well as QRS complex duration at pre-study, and week 2 and 4 and last recovery week. clinical pathology parameters (hematology, coagulation, clinical chemistry, and urinalysis), toxicokinetic parameters, anti-drug antibodies, cytokines, immunophenotypes, organ weights, and macroscopic and microscopic examinations. All analyses, timing and animals are summarized in Table 4 of the manuscript.

Bioanalytical samples were collected on days 1 and 29 at the time of drug administration and after 4, 8, 24, 48, 96 and 120h. Samples were collected following dosing, centrifuged, the resultant serum was frozen immediately at -70°C or colder. SON-1210 serum concentration was determined by a qualified ELISA that employed antibodies against both the IL12 and IL15 domains of SON-1210. The toxicokinetic parameters of SON-1210 were determined by noncompartmental analysis (NCA) using the Phoenix WinNonlin software (version 8.3.4.295).

Anti-Drug Antibodies (IgGs or IgMs) were quantified in monkey sera in two stages starting with a screening assay followed by a confirmatory assay. For the screening stage, sera from the naïve monkeys were screened for the presence of ADAs and the presence of mIL15. If a serum contained mIL15, then this cytokine could disrupt the capture step of the SON-1210 assay, interfering with quantification of the method.

The screening ELISA assay was performed by coating an ELISA plate with SON-1210 and then, after washing and blocking, incubating the wells with 50-fold dilutions of the test sera for the IgG assays and with 20-fold dilutions of the test sera for the IgM assays. After washing, any ADAs that bound to the plate were detected with either HRP-anti-monkey IgG or HRP-anti-human IgM All plates were coated with SON-1210 dissolved in 1X PBS made from tablets (EMD Millipore). The wash buffer was 1X PBS, 0.05% Tween 20 (Amresco).

For the IgG assay, the block buffer was 6% Dry Milk (Bio-Rad) in TBS (pH 8.0; Sigma), and the dilution buffer for sera and the detection antibody was TBS, 6% Dry Milk, 0.1% Tween 20. The

detection antibody was Horseradish Peroxidase (HRP)-Rabbit anti-monkey IgG (Sigma). For the IgM assay, the block buffer and the serum dilution buffer were IgM-Reducing assay diluent (IgM Dilution Buffer; ImmunoChemistry Technologies). The dilution buffer for the detection antibody was TBS, 6% Dry Milk, 0.1% Tween 20. The detection antibody was HRP-goat anti-human IgM mu (SeraCare).

For the confirmation assays, each serum was assayed on the same plate with and without the addition of 12 mg/mL SON-1210. The spiked and unspiked serum samples were incubated for 15 minutes in a polypropylene plate at room temperature and then added to a SON-1210 coated plate as described for the IgG and IgM screening assays. This approach was applied to both pretest (collected prior to SON-1210 treatment, data not shown) or test (collected on Days 15, 35 or 71 following treatment on Days 1, 15 and 29, respectively) samples.

Cytokine samples collection took place on day 1 and 29 at the time of drug administration and after 24, 48, 96 and 120h. Recovery group cytokine samples collection occurred on days 43 and 71. Samples were kept on crushed wet ice until centrifugation carried out within 60 minutes of collection. Samples were centrifuged for 10 minutes in a refrigerated centrifuge (+4°C) at 1200 g. The resulting plasma was split into 2 aliquots (125µL in the first and any residual in the second) and frozen immediately at -70°C. IFNγ, TNF-α, IL-6, IL-8, IL-10, and IL-1β, were multi-plex analyzed using Luminex method in duplicates wherever possible.

Immunophenotyping samples were collected from all animals prior to dosing and then on days 7, 15, 29 and 35. Recovery groups were sampled on days 43 and 71. Samples were transferred at ambient temperature and analyzed using qualified laboratory method Concurrent hematology data were used for the quantification of lymphocyte subset absolute counts. The total lymphocyte absolute counts were taken from the whole blood hematology analysis and applied to the relative lymphocyte percentages obtained by flow cytometry.

Animals were euthanized 35 days after first administration except recovery groups that were sacrificed on day 71. All animals underwent necropsy examination, tissue collection, organ weighting and microscopic histology evaluation.

Toxicokinetic assessment was conducted on samples collected on days 1 and 29 at 0, 4, 8, 24, 48, 96, and 120 hours. SON-1210 quantification was conducted using a combination assay by first capturing the molecule with an IL-15 domain specific antibody and then detecting the quantity of captured material with a biotinylated IL-12 domain specific antibody. hIL-15 Uncoated ELISA Kit (ThermoFisher) provided the anti-IL-15 capture antibody, biotinylated anti-hIL12 detection antibody was purchased from Thermofisher. Plates were coated with the anti-IL15 capture antibody diluted 125 x in coat buffer and, after samples were applied to the plate, SON-1210 was detected with the biotinylated anti-IL12 detection antibody diluted 62.5 x in ELISA diluent. All other steps were followed as outlined in the manufacturer's hIL15 Product Information Sheet. A noncompartmental approach consistent with the subcutaneous route of administration was used to determine the TK parameters. All parameters were generated from individual/composite concentrations of the test article in serum after the final dose.
